# Mental health literacy: a cross-cultural approach to knowledge and beliefs about depression, schizophrenia and generalized anxiety disorder

**DOI:** 10.3389/fpsyg.2015.01272

**Published:** 2015-09-08

**Authors:** Laura Altweck, Tara C. Marshall, Nelli Ferenczi, Katharina Lefringhausen

**Affiliations:** Department of Life Sciences, College of Health and Life Sciences, Brunel University LondonUxbridge, UK

**Keywords:** Mental health literacy, culture, help-seeking, lay help, professional help, schizophrenia, depression, collectivism

## Abstract

Many families worldwide have at least one member with a behavioral or mental disorder, and yet the majority of the public fails to correctly recognize symptoms of mental illness. Previous research has found that Mental Health Literacy (MHL)—the knowledge and positive beliefs about mental disorders—tends to be higher in European and North American cultures, compared to Asian and African cultures. Nonetheless quantitative research examining the variables that explain this cultural difference remains limited. The purpose of our study was fourfold: (a) to validate measures of MHL cross-culturally, (b) to examine the MHL model quantitatively, (c) to investigate cultural differences in the MHL model, and (d) to examine collectivism as a predictor of MHL. We validated measures of MHL in European American and Indian samples. The results lend strong quantitative support to the MHL model. Recognition of symptoms of mental illness was a central variable: greater recognition predicted greater endorsement of social causes of mental illness and endorsement of professional help-seeking as well as lesser endorsement of lay help-seeking. The MHL model also showed an overwhelming cultural difference; namely, lay help-seeking beliefs played a central role in the Indian sample, and a negligible role in the European American sample. Further, collectivism was positively associated with causal beliefs of mental illness in the European American sample, and with lay help-seeking beliefs in the Indian sample. These findings demonstrate the importance of understanding cultural differences in beliefs about mental illness, particularly in relation to help-seeking beliefs.

“We've had a lot of trouble with Western mental health workers…They would do this bizarre thing. They didn't take people out in the sunshine, where you begin to feel better…They didn't involve the whole community…Instead what they did was they took people one at a time into dingy little rooms and had them talk for an hour about bad things that had happened to them.”

Rwandan man (Solomon, 2010)

## Introduction

The Rwandan man's confusion over Western mental health care illustrates how beliefs about mental illness and their treatment can differ across cultures (Jorm, 2000). The World Health Report (World Health Organisation, 2001) revealed that 1 in 4 families worldwide are likely to have at least one member with a behavioral or mental disorder. As the likelihood of coming into contact with someone who suffers from mental illness is high, one would assume that the public would have a comprehensive awareness and understanding of mental disorders. Indeed, greater familiarity with mental illness—i.e., knowledge about mental illness and contact with people with a mental disorder—is associated with more positive beliefs about mental illness (Corrigan et al., 2001). Nevertheless, the majority of the public is unable to recognize and distinguish between different mental disorders (Jorm, 2000; Angermeyer and Dietrich, 2006; Jorm et al., 2006). Large cultural differences exist in this respect, with non-Western populations showing less recognition compared to Western ones (Jenkins, 1988; Ayalon and Arean, 2004; Jorm et al., 2005), yet little research has examined the variables that might drive these cultural differences. The main aim of the present study was to identify the cultural variables that influence knowledge and beliefs about mental illness.

Mental health literacy (MHL) refers to the “knowledge and beliefs about mental disorders which aid their recognition, management or prevention” (Jorm et al., 1997a, p. 184). The concept of MHL is multifaceted and includes: (a) the ability to recognize symptoms of mental illness, (b) knowledge of causes of mental disorders, (c) beliefs that promote recognition and seeking appropriate help, and knowledge of (d) lay sources of help and (e) professional sources of help (Jorm et al., 1997a). Individuals who are confronted with symptoms of mental illness—by developing a disorder themselves or by coming into contact with someone who has—will endeavor to manage these and an individual's approach will depend on their mental health literacy. In other words better knowledge and more positive beliefs about mental illness will positively alter patterns of help-seeking as well as responses to treatment (Jorm, 2000, 2011; ten Have et al., 2010).

Most studies examining MHL have focused on depression and schizophrenia (Jorm, 2000; Angermeyer and Dietrich, 2006). We considered these disorders in the present study, but we additionally examined generalized anxiety disorder (GAD) because the prevalence worldwide for this disorder is also high [The World Health Report (World Health Organisation, 2001); Kessler et al., 2005] and to the best of our knowledge MHL has not been previously examined in relation to GAD.

**Figure 1:**
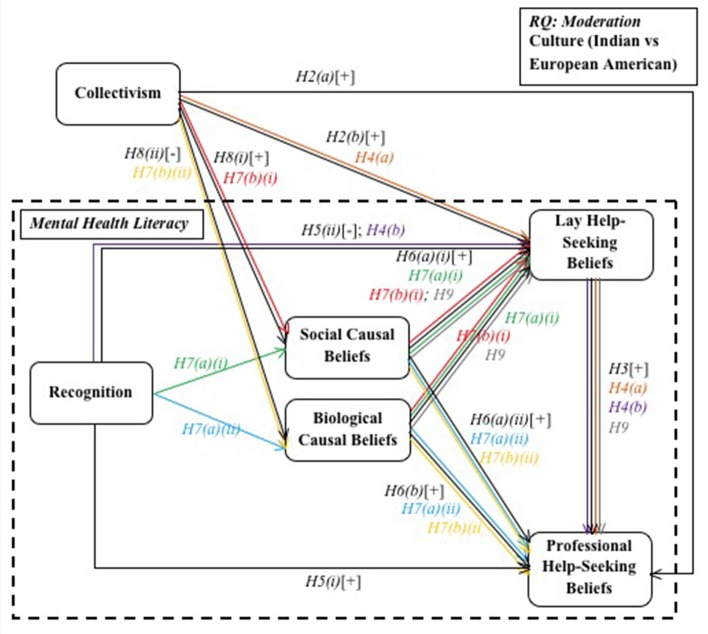
**Proposed mental health literacy model**.

Further, MHL, on a holistic level, has generally been studied from a qualitative approach (Jorm et al., 1997a,b,c; Jorm, 2000, 2011), whereas individual aspects of MHL have been studied quantitatively (Atkinson et al., 1991; Tata and Leong, 1994; Yeh, 2002; Kuo et al., 2007). To the best of our knowledge a combined model of MHL has not been tested quantitatively. In the present study we proposed and tested a mediational model of MHL moderated by cultural background (see Figure 1). Below we outline the proposed associations within the model.

### MHL and culture

Cross-cultural studies show that, on average, compared to non-Western populations Western ones show greater medical knowledge of mental disorders and lesser stigma toward mental illness (Jorm, 2000; Angermeyer and Dietrich, 2006). In the present study, we compared individuals of European descent living in the USA with Indians living in India (going forward referred to as European Americans and Indians respectively). We chose these two particular cultures, first, because they differ in their availability of mental health facilities. The World Health Organisation (2011a,b) reported that per 100,000 people in India, there are 0.33 mental health outpatient facilities, 0.30 psychiatrists, and 0.05 psychologists, whereas in the USA these figures were 1.95, 7.79, and 29.0, respectively. As clinical mental health facilities are more accessible in the USA, they are seen as viable solutions to managing symptoms of mental health. Further, because close to 75% of the Indian population resides in rural areas—lacking satisfactory primary health care—religious leaders and traditional healers are seen as the main resource for treatment of mental disorders (Khandelwal et al., 2004).

We also chose these two cultures because stigma toward mental illness significantly differs between Western and non-Western cultures, with Asian and African populations showing higher rates of mental illness stigma (Cooper-Patrick et al., 1997; Whaley, 1997; Angermeyer et al., 2004; Mishra et al., 2009; Vijayalakshmi et al., 2013). In particular, mental illness stigma may present as a preference of distancing oneself from people with mental illness (Shokoohi-Yekta and Retish, 1991; Adewuya and Fwacp, 2005; Vijayalakshmi et al., 2013) or the belief that people with mental illness are different and inferior (Shokoohi-Yekta and Retish, 1991), dangerous (Whaley, 1997; Kurihara et al., 2000; Barke et al., 2011), or violent (Kurihara et al., 2000; Anglin et al., 2006).

Stigma toward people with a mental illness in particular in India has been shown to be high (Thara and Srinivasan, 2000; Weiss et al., 2001; Abdullah and Brown, 2011; Vijayalakshmi et al., 2013). In the Indian culture, individuals with a mental disorder are often shunned by their community (Shankar et al., 2006). Mental illness may pose as a hindrance to being able to fulfill one's roles and duties and is thought to affect family relationships—for example, making it difficult to find a marriage partner for oneself or family members (Weiss et al., 2001; Shankar et al., 2006). To the best of the authors' knowledge, MHL has not been compared cross-culturally in India and the USA. Thus, we proposed the following research question:

How will participants' cultural background moderate the MHL model?

While a number of studies have compared MHL cross-culturally (Jorm, 2000; Angermeyer and Dietrich, 2006), scarcely any literature has examined reasons for cultural differences. Hence, the present study aimed to explain cross-cultural variation in MHL by investigating collectivism. This cultural variable denotes a priority to group goals and needs, which results in a strong group- or social identity (Shulruf et al., 2007). In many non-Western cultures, in-group harmony and cohesion is highly valued (Imada and Yussen, 2012). On an individual level, individuals in collectivist cultures are more likely to view themselves as interdependent as well as act according to their group membership and the context they are in Markus and Kitayama (1991). This heightened sense of group identity and interdependence is counterbalanced by individuals' assurance that they will be looked after by their in-group (Hofstede, 1980, 2001). Further, norms, customs and duties in collectivist cultures are highly valued and closely followed (Lykes and Kemmelmeier, 2014). Hofstede (1980, 2001) found that non-Western cultures such as India yielded some of the highest scores for collectivism, whereas Western cultures like the USA scored lowest on this dimension. Below we outline how collectivism may be associated with the aspects of MHL—recognition, causal beliefs, lay help-seeking beliefs, and professional help-seeking beliefs.

### Help-seeking beliefs

#### Professional help-seeking beliefs

The majority of the literature investigating beliefs about help-seeking for mental disorders has focused on seeking professional as opposed to lay help (Tata and Leong, 1994; Kuo et al., 2007; ten Have et al., 2010). Professional help may include seeing a general practitioner, psychologist, counselor or psychiatrist, or going to a mental health clinic. Jorm et al. (1997c) found that the public tends to prefer seeking help from more general health practitioners compared to specialist help. In the present study we developed a new professional help-seeking beliefs measure and tested its construct validity (the degree to which a scale's items reflect the construct being measured and encompasses the entirety of the construct; Field, 2009). We hypothesized that:

*H1(a): The professional help-seeking beliefs measure developed in the current study will be valid (i) across cultures and (ii) between mental disorders*.

Collectivist individuals tend to value in-group relations and seeking advice from the in-group (Hofstede, 1980, 2001; Shulruf et al., 2007), indicating that collectivists would be more inclined to seek help in general, both from lay and professional sources. Along these lines, Kuo and associates (2007) found that individuals who reported greater interpersonal harmony—belief of social cooperation, familial support and unity—were more likely to positively endorse seeking professional psychological help. They inferred that individuals endorsing interpersonal harmony perceived the psychological relationship as nurturing, safe, and trustworthy. Collectivism is linked with an interdependent self-construal—the perception that the self is embedded in important social relationships (Markus and Kitayama, 1991). Yeh (2002) investigated interdependent self-construals in relation to beliefs about going to counseling and found that greater endorsement of interdependent self-construal predicted more positive beliefs about seeking professional help. These results indicate that individuals who placed greater value on relationships and connectedness tended to have more positive beliefs about professional psychological help-seeking. Thus, it is viable to propose that this association would translate to the cultural-level, namely, that greater collectivism would be associated with greater endorsement of seeking professional help for symptoms of mental illness. Indeed, Tata and Leong (1994) found that greater endorsement of collectivism was related to more positive beliefs about professional psychological help-seeking. A limitation with this study is that it conceptualized collectivism as one pole along a unipolar individualism-collectivism dimension. However, research generally finds that individualism and collectivism are two orthogonal dimensions (see Freeman and Bordia, 2001), indicating that individuals can be both individualist and collectivist at the same time. The present study conceptualized collectivism as orthogonal to individualism, and so we predicted that:

*H2(a): Greater collectivism will be associated with more positive professional help-seeking beliefs*.

#### Lay help-seeking beliefs

As only a minority of individuals with symptoms of mental illness seek professional help, it is even more important for them to seek help from other sources (Jorm, 2000). Indeed, individuals with symptoms of mental illness look for support from a wide range of informal sources (Cooper-Patrick et al., 1997; Van Hook, 1999; Chadda et al., 2001; Shankar et al., 2006; Penny et al., 2009). Individuals with a mental illness draw informal support primarily from in-group members (i.e., family members, friends, or religious leaders; Daly et al., 1995; Van Hook, 1999). In the present study we consider lay help for mental illness as seeking help or advice from a non-medical professional—e.g., family, friends, spiritual leader—as well as engaging in an activity with the aim to reduce symptoms of mental illness—e.g., taking herbs, doing exercise or going on holiday. In the present study we developed a new lay help-seeking beliefs measure and hypothesized that:

*H1(b): The lay help-seeking beliefs measure developed in the current study will be valid (i) across cultures and (ii) between mental disorders*.

Jorm et al. (1997c) compared members of the public with medical professionals in their beliefs about mental illness and found that the public tended to rate lay help—close friends, herbalists or taking vitamins or minerals—as more helpful. Jorm (2000) reported that individuals with a mental disorder were more likely to seek professional help if their friends and family members positively endorsed this. We therefore proposed that:

*H3: More positive lay help-seeking beliefs will significantly predict more positive professional help-seeking beliefs*.

Van Hook (1999) further found that preference for type and degree of use of lay help differs cross-culturally. Because collectivists tend to seek help and support from the in-group, it is reasonable to surmise that more collectivist individuals would utilize lay sources of help to a greater extent. Indeed, in more collectivist cultures mental illness is perceived as a communal concern; for example, in the Filipino culture the whole family identifies as having a mental illness (Sanchez and Gaw, 2007). Further, in the Indian culture, the primary responsibility for the care of a person with mental illness lies with the family, who will make crucial decisions about treatment and care (Khandelwal et al., 2004). Thus, we hypothesized that:

*H2(b): Greater collectivism will be associated with more positive lay help-seeking beliefs*.

Furthermore, individuals faced with symptoms of mental illness seek support from a range of informal sources before seeking professional help (Cooper-Patrick et al., 1997; Van Hook, 1999). Patients who have sought help for their mental illness report that if a friend or family member endorsed professional help they were more likely to seek support from a professional (Penny et al., 2009). Similarly, in rural areas in India, traditional healers are seen as the main source of help for physical and mental health issues in a village and traditional healers would make the decision to seek a medical professional when they felt it necessary (Shankar et al., 2006). This indicates a positive relationship between lay and professional help-seeking beliefs. Recall that we proposed that collectivism positively predicted both professional help-seeking beliefs and lay help-seeking beliefs; we further proposed that:

*H4(a): The MHL model will display indirect effects between collectivism and professional help-seeking beliefs through lay help-seeking beliefs*.

### Recognition

Another aspect of MHL is recognition of mental disorders, which tends to be poor amongst members of the public (Jorm et al., 1997a; Jorm, 2000; Lauber et al., 2003; Angermeyer and Dietrich, 2006; Dahlberg et al., 2008). For example, schizophrenia is often incorrectly associated with a split conscience or personality (Angermeyer and Matschinger, 1999), while symptoms of depression are sometimes perceived as a physical disorder such as a virus, nutritional deficiency or cancer (Jorm et al., 1997a).

Cultural differences are evident in the public's recognition of mental disorders, with European individuals being significantly better at recognizing symptoms of mental disorders (65–78%) than their Asian and African counterparts (20–26%; Jenkins, 1988; Ayalon and Arean, 2004; Jorm et al., 2005). Vijayalakshmi et al. (2013) found that 81% of a rural, lay, Indian sample reported that they had no previous contact with mental illness. However, as the Indian national prevalence rate of mental illness is estimated at 5.8% (World Health Organisation and Wonca, 2014), Vijayalakshmi et al. (2013) findings indicate a low level of awareness and psychiatric knowledge about mental disorders.

Recognition of mental illness is further linked with the other aspects of the MHL model. Better knowledge about mental disorders in general is a good indicator of knowledge about treatment options and beliefs about causes of mental disorders (Jorm et al., 1997b; Lauber et al., 2003; Wright et al., 2007). Labeling symptoms as a mental illness is associated with identifying the need to seek professional help and, indeed, greater endorsement of seeking help from a professional (Lauber et al., 2003; Wright et al., 2007). Further, better recognition of mental illness is related to lesser endorsement of lay coping strategies—such as drug use (Wright et al., 2007). Labeling symptoms as a mental disorder may activate a schema that outlines the type of action to take (Jorm, 2011), that is, better knowledge about mental disorders would encourage a preference for professional compared to lay help. Thus we hypothesized:

*H5: Better recognition of mental disorders will predict (i) more positive professional help-seeking beliefs, and (ii) more negative lay help-seeking beliefs*.

Also recall that the literature has indicated that there is a strong positive relationship between lay and professional help-seeking beliefs (Cooper-Patrick et al., 1997; Van Hook, 1999; Shankar et al., 2006; Penny et al., 2009). Therefore, we also hypothesized that:

*H4(b): The MHL model will display indirect effects between recognition and professional help-seeking beliefs through lay help-seeking beliefs*.

### Causal beliefs

A further facet of MHL concerns beliefs about the causes of symptoms of mental disorders. Psychopathological models draw on social and biological factors when explaining causes of mental disorders. In the present study we developed a social and biological causal beliefs measure and hypothesized that:

*H1(c): The social and biological causal beliefs measure developed in the current study will be valid (i) across cultures and (ii) between mental disorders*.

Greater endorsement of both biological and social causes of mental illness reflects better knowledge of mental illness and therefore identification of the need to seek help from a professional (Atkinson et al., 1991; Jorm et al., 1997b; Williams and Healy, 2001; Chen and Mak, 2008). In line with this, Chen and Mak (2008) investigated the relationship between beliefs about causes and professional help-seeking for symptoms of mental illness and found that greater endorsement of social and biological causal beliefs was positively related to the likelihood of seeking professional help. To the best of our knowledge, the relationship between causal beliefs of mental illness and beliefs about lay help has not been examined. We propose that individuals who believe that mental illness is due to social causes would also be more likely to endorse reaching out to the social environment to manage these symptoms. On the other hand, individuals who believe that mental illness has biological causes would see lay help as an irrelevant vs. professional help as a relevant source of knowledge. Hence, we predicted that:

*H6(a): Individuals who more strongly endorsed social causal beliefs would hold more positive (i) lay and (ii) professional help-seeking beliefs*.

*H6(b): Individuals who more strongly endorsed biological causal beliefs would hold more positive professional help-seeking beliefs*.

Further, Jorm and associates (1997b) found an association between recognition and causal beliefs of mental illness. For instance, they found that participants who correctly recognized symptoms of schizophrenia were more likely to cite genetic or inherited factors as causes. As the literature has shown that recognition would be a strong positive predictor of causal beliefs and we previously predicted that recognition would be strongly positively associated with lay and professional help-seeking beliefs, we further hypothesized that:

*H7(a): The MHL model will show significant indirect effects between recognition and (i) lay and (ii) professional help-seeking beliefs through causal beliefs*.

Endorsement of causal beliefs also varies between cultures (Narikiyo and Kameoka, 1992; Sheikh and Furnham, 2000); for example, individuals from some Asian or African cultures may attribute causes of mental disorders to supernatural phenomena (Razali et al., 1996; Sheikh and Furnham, 2000; Ohaeri and Fido, 2001; Suhail, 2005), whereas in Western cultures such attributions are less prevalent (Angermeyer and Matschinger, 1999). The public worldwide tends to favor social causes of mental illness to biological ones (Jorm, 2000; Beck et al., 2003; Angermeyer and Dietrich, 2006), yet cultural differences in this respect are also evident (Narikiyo and Kameoka, 1992; Schnittker et al., 2000; Sheikh and Furnham, 2000; Dietrich et al., 2003; Speller, 2005). For instance, Narikiyo and Kameoka (1992) found that Japanese American students at American universities reported greater agreement with social causes and lesser endorsement of biological causes for symptoms of mental illness compared to their European American counterparts. Similarly, Dietrich and associates (2003) investigated causal beliefs of mental disorders cross-culturally and found that Russian and Mongolian participants tended to attribute the causes of mental illness significantly more to the family than their German counterparts. Characteristics of collectivism would indicate that collectivists would be more likely to attribute causes of mental illness to the community. Indeed, both Narikiyo and Kameoka's (1992) and Dietrich and associates' (2003) findings support this notion, because, Russians and Mongolians compared to Germans and, similarly, Japanese Americans compared to European Americans, tend to be more collectivist (Hofstede, 1980, 2001). Thus, we hypothesized that:

*H8: Greater collectivism will be associated with greater endorsement of (i) social and less endorsement of (ii) biological causal beliefs*.

Recall that we predicted that social causal beliefs would be significantly positively associated with lay and professional help-seeking beliefs, while biological causal beliefs more would be significantly positively related to professional help-seeking beliefs and significantly negatively related to lay help-seeking beliefs. We therefore also hypothesized that:

*H7(b): The MHL model will show significant indirect effects between collectivism and (i) lay and (ii) professional help-seeking beliefs through causal beliefs*.

Finally, also recall that the literature indicated a strong positive relationship between lay and professional help-seeking beliefs (Cooper-Patrick et al., 1997; Van Hook, 1999; Shankar et al., 2006; Penny et al., 2009). Therefore, we also hypothesized that

*H9: The MHL model will show significant indirect effects between causal beliefs and professional help-seeking beliefs through lay help-seeking beliefs*.

## Method

### Ethics statement

Ethical approval was obtained from the Brunel University Psychology Research Ethics Committee. Participants provided written informed consent at the beginning of the survey and all responses were confidential.

### Participants and procedure

The study was conducted online through a survey-building website. Participants were invited to take part in a study about knowledge and beliefs about mental health. All materials were in English only. A hyperlink to the survey was distributed through a London university's intranet site, social networking sites, and through Amazon's Mechanical Turk, where participants were offered $0.30 upon completion of the survey (IP addresses were inspected to ensure there were no multiple entries).

We invited European Americans currently living in the USA (*N* = 100) and Indians currently living in India (*N* = 108) to participate in this study. Ipeiriotis (2010) found that 52% of US-based MTurk workers have a household income between $25,000–75,000/year while 55% Indian MTurk workers declared an income of $10,000/year. MTurk workers from India are more often male, younger, more highly educated and more likely to report relying on the income from MTurk than their counterparts from the USA (Ross et al., 2009). In regards to the present sample we conducted Chi Square and *t*-tests of demographic variables with culture as a group variable to identify mean differences (see Table S1). The Indian sample was significantly younger, more educated and was made up of significantly more men than the European American sample. The majority of the Indian sample identified as Hindu, while the European American sample was divided between identifying as Christian and non-religious.

### Measures

#### Collectivism

This was measured with the collectivism subscale of Sivadas et al. (2008) 14-item short-form of the vertical-horizontal collectivism-individualism scale. Items were rated on a 5–point Likert scale ranging from 1 (strongly disagree) to 5 (strongly agree). A sample item includes “My happiness depends very much on the happiness of those around me.” To increase reliabilities, we collapsed across the vertical and horizontal dimensions so that 8 items measured collectivism (α = 0.84).

#### Vignettes

Participants were asked to read three vignettes, each describing a person with symptoms of depression (Jorm et al., 1997a), schizophrenia (Jorm et al., 1997a) or GAD (Leitschuh, 2008). The vignettes were slightly shortened for the present study. See the modified vignettes in the Data Sheet 1.

#### Recognition

After reading each vignette, participants answered the question, “What do you think is going on with the person?” to measure their recognition of the mental disorder presented in each vignette. If participants gave non-mental health related responses they received a score of “1,” any mental illness-related responses (e.g., “mental disorder”) scored “2,” and naming the particular mental disorder displayed in the vignette scored “3” (i.e., “depression” in relation to the depression vignette). Reliabilities were α = 0.75 for all mental disorders and α = 0.89, 0.68, and 0.49 for schizophrenia, depression and GAD, respectively.

#### Item selection: Causal beliefs, lay help-seeking beliefs and professional help-seeking beliefs measures

After reading the vignettes, participants were also presented the causal beliefs, lay help-seeking beliefs and professional help-seeking beliefs measures developed in the present study. We chose the items for these measures from studies that had examined MHL using qualitative approaches (Jorm et al., 1997a, 2006; Jorm, 2000; Dietrich et al., 2003; Ayalon and Arean, 2004; Peluso and Blay, 2004; Angermeyer and Dietrich, 2006; Wright et al., 2007; Dahlberg et al., 2008). See Table S2 of for all items of the developed measures.

The causal beliefs measure posed the following question: “To what extent do you think that the following could explain the person's behavior?” The 6-item measure was rated along a 5-point Likert scale ranging from 1 (Completely explains the behavior) to 5 (Does not explain the behavior). Items of the causal beliefs measure were reverse-coded such that higher scores represent greater agreement that causes explain the person's behavior.

The lay help-seeking beliefs and professional help-seeking beliefs measures posed the following question: “To what extent do you think it would be helpful or harmful for your friend to… ?.” Participants were asked to rate items of both the lay and professional help-seeking beliefs measures along a 7-point Likert scale that ranged from 1 (Very helpful) to 7 (Very harmful). Items of the lay and professional help-seeking beliefs measures were reverse-coded such that higher scores represent beliefs of greater helpfulness. See Table S3 for scale reliabilities, means, and standard deviations for all of the items of the causal beliefs, lay help-seeking beliefs, and professional help-seeking beliefs measures.

### Data analysis

For our newly developed causal beliefs, lay help-seeking beliefs, and professional help-seeking beliefs measures, we examined measurement equivalence or invariance (the degree a measure functions the same way across cultures; French and Finch, 2006; Gere and MacDonald, 2013). Failure to establish measurement equivalence threatens the validity of conclusions drawn in cross-cultural research (Singh, 1995; Diamantopoulos and Papadopoulos, 2010). Evaluation of measurement equivalence is rooted in classical test theory of true and error scores, which enables the evaluation of a measure's reliability and validity (Vandenberg and Lance, 2000; Diamantopoulos and Papadopoulos, 2010).^1^

A common method of examining measurement equivalence or invariance is by means of multi-group confirmatory factor analysis (French and Finch, 2006; Diamantopoulos and Papadopoulos, 2010; Gere and MacDonald, 2013). Measurement equivalence or invariance testing involves the comparison of increasingly more restrictive models by constraining them to be equal across groups (French and Finch, 2006; Gere and MacDonald, 2013).

We conducted multiple-group factor analyses for each measure separately. Each construct—causal beliefs, lay help-seeking beliefs, and professional help-seeking beliefs—was modeled as a latent variable with the individual scale items representing observed variables (see Table S4 for zero-order correlations between items for each sample and mental disorder). Cross-cultural invariance of the measurement model was tested in both the overall measurement model as well as individual loadings. Items or models were considered as invariant when the chi-square difference test was non-significant. If items did not hold cross-cultural invariance, we tested further models where invariant items were removed one by one until all remaining items met cultural equivalence. At each step we removed items or paths with the highest *p*-value in both cultural groups.

For the statistical analyses we employed AMOS 18. As AMOS 18 requires data without missing values, we used the expectation-maximization estimation method to deal with the missing values in our data set (Dempster et al., 1977). The expectation-maximization algorithm is based on the assumption that values are missing at random and it is recommended that no more than 2% of the data set is missing (Dempster et al., 1977; Schafer, 1997); both were the case in the present data set (Little's MCAR test: *p* > 0.05, *t*-tests: *p* > 0.05, *N*_missing values_ < 1.85%). Then the algorithm computes maximum likelihood estimates. We used SPSS 20 to input items into the expectation-maximization algorithm. To increase the power of our data we input items associated with one particular subscale at time, as this increases the correlations between items (Dempster et al., 1977).

We followed Kline's (2011) guidelines to evaluate model fit: non-significant chi-square value, a comparative fit index (CFI) greater than.90, a root mean square error of approximation (RMSEA) of 0.08 or less, and a standardized root mean square residual (SRMR) of 0.10 or less. With large samples, it is unrealistic for the chi-square value to be non-significant, therefore we relied more heavily on the other indices. To compare the model fit between models we employed the chi-square difference test (Kline, 2011).

## Results

### Hypothesis 1(a): Validating the professional help-seeking beliefs measure

**Figure 2:**
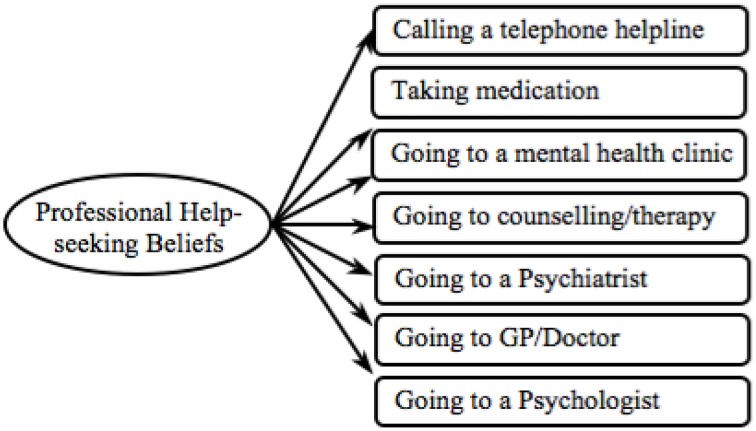
**Proposed professional help-seeking beliefs model**.

First we examined the validity of the professional help-seeking beliefs measure across (i) cultures and (ii) mental disorders. We performed multi-group CFA with culture—European Americans vs. Indians—as the group-variable, and tested this measure separately for each mental disorder. The model displayed in Figure 2 was tested.

First the professional help-seeking beliefs measure was examined in relation to the depression data. The model proved to be a good fit (see Depression Model 1 in Table 1) and the overall model loaded equivalently across cultures (*p*s > 0.05). Yet *seeing a psychologist* loaded more strongly in the European American sample while *going to counseling or therapy* loaded more strongly in the Indian sample (see Depression in Table S5). Thus, we tested subsequent models by removing the most invariant item. Items were eliminated in the following order: *going to counseling or therapy* then *seeing a psychologist*. The refined model held an excellent model fit (see Depression Model 3 in Table 1), which can be employed for cross-cultural comparison in relation to depression.

**Table 1:** **Model fit indices for the professional help-seeking beliefs measure**.

					**RMSEA**						
**Model**	**χ^2^**	**df**	***p***	**CFI**		**LB**	**HB**	**SRMR**	**Δχ^2^**	**df**	***p***
**DEPRESSION**											
Unconstrained	43.64	28	0.03	0.98	0.05	0.02	0.08	0.04			
Model 1	53.69	35	0.02	0.97	0.05	0.02	0.08	0.07	10.05	7	0.19
Model 2	17.52	18	0.49	1.00	0.021	< 0.001	0.062	0.06	43.23	18	0.0007
Model 3	13.78	10	0.18	0.99	0.030	< 0.001	0.075	0.06	3.74	8	0.88
Model—final	2.20	3	0.53	1.00	< 0.001	< 0.001	0.104	0.03	20.18	13	0.09
**SCHIZOPHRENIA**											
Unconstrained	61.00	28	0.001	0.94	0.08	0.06	0.11	0.06			
Model 1	87.10	35	0.001	0.92	0.09	0.061	0.106	0.07	26.10	7	0.0005
Model 2	47.51	24	0.003	0.95	0.069	0.039	0.098	0.07	39.59	11	0.00004
Model 3	26.93	15	0.03	0.10	0.062	0.020	0.099	0.06	20.58	9	0.01
Model 4	9.05	8	0.34	0.10	0.025	0.001	0.088	0.03	17.88	7	0.01
Model—final	0.56	3	0.91	1.00	< 0.001	< 0.001	0.05	0.01	8.49	5	0.13
**GAD**											
Unconstrained	72.68	28	0.001	0.92	0.09	0.06	0.11	0.07			
Model 1	80.94	35	0.001	0.91	0.08	0.06	0.10	0.09	8.26	7	0.31
Model 2	70.82	24	0.001	0.89	0.10	0.07	0.12	0.08	10.12	11	0.52
Model 3	40.17	15	0.001	0.90	0.09	0.06	0.13	0.05	30.65	9	0.0003
Model 4	18.81	8	0.02	0.94	0.08	0.03	0.13	0.05	21.36	7	0.003
Model—final	3.79	3	0.29	0.99	0.04	< 0.001	0.13	0.03	15.02	5	0.01

Next we examined the professional help-seeking beliefs measure in relation to the schizophrenia data and found that the model had a good fit (see Schizophrenia Model 1 in Table 1). However, the model did not load equally between cultures [χ^2^(7) = 22.09, *p* = 0.002]. On closer, inspection several items did not load invariantly (see Schizophrenia in Table S5). Thus, we tested further models by removing the most invariant item at each step. Items were removed in the following order: *see a psychologist, go for counseling or therapy* and *see a GP/doctor*. The refined model held an excellent model fit (see Schizophrenia Model 4 in Table 1), which can be employed for cross-cultural comparison in relation to schizophrenia.

Finally we tested the original model in regards to the GAD data. The overall model loaded equivalently across cultures (*p* > 0.05), however it proved to be a poor fit (see GAD Model 1 in Table 1). On closer, inspection several items did not load invariantly (see GAD in Table S5). Thus, we tested further models eliminating the most invariant item at each step. Items were removed in the following order: *go for counseling and/or therapy, see a psychiatrist* and *see a GP/doctor*. The refined model held a good model fit (see GAD Model 4 in Table 1), which can be employed for cross-cultural comparison in relation to GAD.

**Figure 3:**
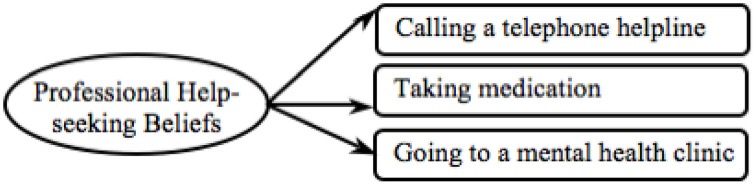
**Final professional help-seeking beliefs model**.

In order to be able to compare findings between mental disorders we tested a model that encompassed only the items that were culturally invariant across all three mental disorders (see Figure 3). The final model showed an excellent fit in relation to all three mental disorders (final Models in Table 1), confirming both H1(a)(i) – cross-cultural validity—and H1(a)(ii) – validity across mental disorders. Thus, in the following analyses we employed the final professional help-seeking beliefs measure displayed in Figure 3.

### Hypothesis 1(b): Validating the lay help-seeking beliefs measure

**Figure 4:**
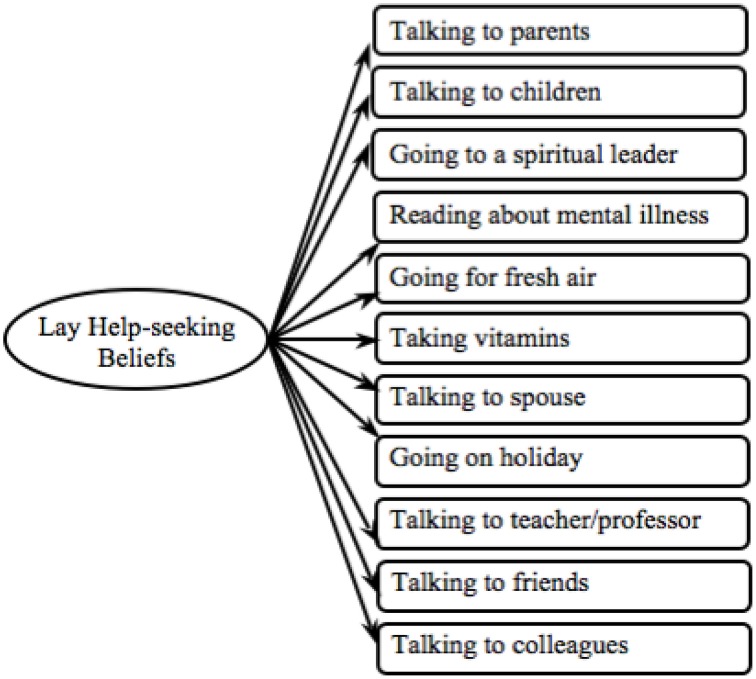
**Proposed lay help-seeking beliefs model**.

We used the same approach to test the validity of the lay help-seeking beliefs measure between (i) cultures and (ii) mental disorders (see Figure 4). First the model was examined in relation to the depression data for which the model proved to be an adequate fit (see Depression Model 1 in Table 2). All loadings between observed variables and latent variables were invariant and significant in both cultures (see Depression in Table S6). Thus, this measure can be employed for cross-cultural comparison in relation to the depression data.

**Table 2:** **Model fit indices for the lay help-seeking beliefs measure**.

					**RMSEA**						
**Model**	**χ^2^**	**df**	***p***	**CFI**		**LB**	**HB**	**SRMR**	**Δχ^2^**	**df**	***p***
**DEPRESSION**											
Unconstrained	214.51	88	0.001	0.88	0.08	0.07	0.1	0.08			
Model 1	220.57	98	0.001	0.89	0.08	0.06	0.09	0.08	6.06	10	0.81
Model—final	142.86	79	0.001	0.93	0.06	0.05	0.08	0.07	77.71	19	< 0.00001
**SCHIZOPHRENIA**											
Unconstrained	280.19	88	0.001	0.83	0.10	0.09	0.12	0.08			
Model 1	299.64	98	0.001	0.82	0.10	0.09	0.11	0.09	19.45	10	0.03
Model—final	212.14	79	0.001	0.86	0.09	0.08	0.10	0.06	87.5	19	< 0.00001
**GAD**											
Unconstrained	380.03	88	0.001	0.70	0.13	0.11	0.14	0.12			
Model 1	387.34	98	0.001	0.71	0.12	0.11	0.13	0.13	7.31	10	< 0.00001

**Figure 5:**
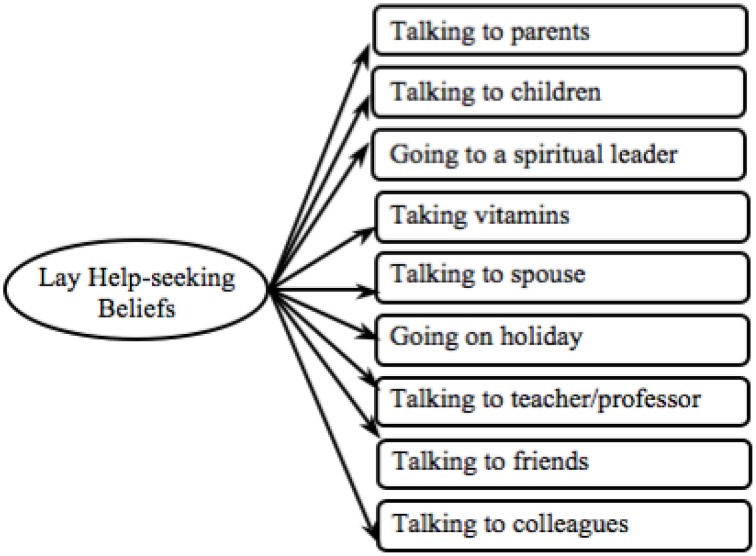
**Final lay help-seeking beliefs model**.

Next we tested the lay help-seeking beliefs measure in relation to the schizophrenia data and found that the model was a poor fit (see Schizophrenia Model 1 in Table 2). Indeed the model did not load equally between cultures [χ^2^(10) = 19.45, *p* = 0.04]. The item *fresh air* did not load equally between groups (see Schizophrenia in Table S6) and a refined model without this item was tested (see Figure 5). The refined model was invariant across cultures, with all observed variables loading equivalently onto the latent variables (*p*s > 0.05). The refined model held an adequate model fit and had significantly improved (see Schizophrenia Model final in Table 2).

Next we tested the original model in regards to the GAD data. All latent variables significantly mapped onto the latent variable and all observed variables loaded equivalently onto the latent variable (see GAD in Table S6). Overall, however, the model fit was poor (see GAD Model 1 in Table 2). As H2(b)(ii) – testing validity across mental disorders—was only partially confirmed the lay help-seeking beliefs measure can only be used to analyze data concerning depression and schizophrenia and not GAD.

In order to be able to compare the lay help-seeking beliefs measure between mental disorders, we used the refined model developed from the schizophrenia data (see Figure 5) and tested this with the depression data. Confirming H1(b)(i) – establishing cross-cultural validity—we found a good model fit, which also significantly improved (see Depression Model final in Table 2). In all following analyses, we used the final lay help-seeking beliefs model (see Figure 5).

### Hypothesis 1(c): Validating the social and biological causal beliefs measure

**Figure 6:**
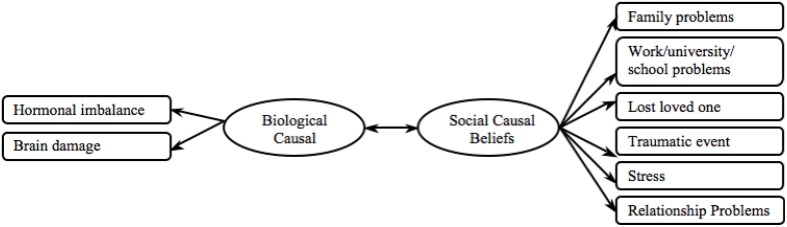
**Proposed causal beliefs model**.

We employed the same approach to examine the validity of the social and biological causal beliefs measure (i) cross-culturally and (ii) between mental disorders. See Figure 6 for an outline of the proposed causal beliefs measure.

First we tested the measure in regards to the depression data. The measurement model was an adequate fit (see Depression Model 1 in Table 3). All loadings of the observed variables significantly mapped onto their respective latent variables in both cultural groups and held cross-cultural equivalence (see Depression in Table S7). However the correlation between biological and social causal beliefs significantly differed between cultures, being significantly, positively correlated in the European American sample while trending toward significance in the Indian sample (see Depression in Table S7). Thus this measure can be used for analyses within a cultural group, but cannot be utilized for cross-cultural comparison of biological and social causal beliefs.

**Table 3:** **Model fit indices for the causal beliefs measure**.

					**RMSEA**						
**Model**	**χ^2^**	**df**	***p***	**CFI**		**LB**	**HB**	**SRMR**	**Δχ^2^**	**df**	***p***
**DEPRESSION**											
Unconstrained	86.78	38	< 0.001	0.93	0.08	0.06	0.10	0.04			
Model 1	97.85	47	< 0.001	0.93	0.072	0.05	0.09	0.07	11.07	9	0.27
Model—final	2.40	3	.49	1.00	< 0.001	< 0.001	0.11	< 0.02	95.45	44	0.00001
**SCHIZOPHRENIA**											
Unconstrained	99.64	38	< 0.001	0.90	0.09	0.07	0.11	0.07			
Model 1	134.69	47	< 0.001	0.85	0.10	0.08	0.11	0.09	35.05	9	0.00006
Model 2	102.11	34	< 0.001	0.86	0.10	0.08	0.12	0.09	32.58	13	0.002
Model 3	72.79	15	< 0.001	0.86	0.137	0.106	0.17	0.08	29.32	19	0.06
Model 4	40.60	8	< 0.001	0.89	0.14	0.10	0.19	0.08	32.19	7	0.00004
Model—final	1.11	3	0.78	1.00	< 0.001	< 0.001	0.09	0.01	39.49	5	< 0.00001
**GAD**											
Unconstrained	112.421	38	< 0.001	0.78	0.10	0.08	0.12	0.09			
Model 1	126.042	47	< 0.001	0.76	0.09	0.07	0.11	0.09	13.62	9	0.44

Next we tested the causal beliefs measure in regards to the schizophrenia data and found that the model fit was poor (see Schizophrenia Model 1 in Table 3). Inspection of individual item loadings showed that all loadings significantly mapped onto their respective latent variables in both cultural groups, except for the biological causal beliefs items *brain damage* and *hormonal imbalance* in the European American sample (see Schizophrenia in Table S7). Further, only the items *traumatic event, family problems*, and *stress* were cross-culturally invariant; all other items loaded significantly more strongly in the European American than in the Indian sample (see Schizophrenia in Table S7). We therefore tested subsequent models by removing the most invariant item at each step. Items were removed in the following order: *work/university/school problems, brain damage, hormonal imbalance, relationship problems*, and *lost a loved one*. The removal of individual items significantly improved model fit and the final model held an excellent model fit (see Schizophrenia Model final in Table 3). The final causal beliefs measure can be utilized cross-culturally in relation to the schizophrenia data.

Finally, we tested the causal beliefs measure in regards to the GAD data. All individual observed variables significantly loaded onto the latent variable and were equivalent across cultures (see GAD in Table S7). However, the model fit was poor (see GAD Model 1 in Table 3). Therefore, this measure can therefore not be used in regards to GAD data.

**Figure 7:**
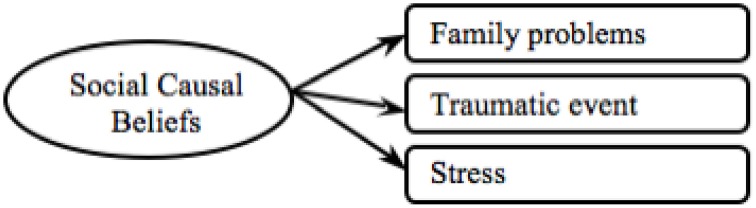
**Final social causal beliefs model**.

In order to compare findings between mental disorders we employed the refined model developed from the schizophrenia data and tested this with the depression data. We found an excellent model fit and found this to be a significant improvement (see Depression Model final in Table 3). Thus, we conclude that Hypothesis 1(c)(i) – establishing cross-cultural validity—was confirmed as the final causal beliefs measure was cross-culturally valid (see Figure 7). In all of the following analyses the final causal beliefs scale was used. As only the social causal belief items were found valid, going forward we refer to this measure as social causal beliefs measure.

Also Hypothesis 1(c)(ii) – establishing validity between mental disorders—was only partially supported, as items of the causal beliefs scale were only valid in relation to the depression and schizophrenia data.

As the social causal beliefs and the lay help-seeking beliefs measures held poor model fit in regards to the GAD data, only the depression and schizophrenia data was used in the following analyses. In all following analyses we employed social causal beliefs, lay help-seeking beliefs and professional help-seeking beliefs as the latent variables, with both the depression and schizophrenia items loading on the respective constructs.

### Hypotheses 2, 3, 5, 6, 8: Model testing

Normality tests (*p* > 0.05) and inspection of the histograms showed that the professional help-seeking beliefs, lay help-seeking beliefs and social causal beliefs constructs were normally distributed in both cultural samples. Recognition showed negative kurtosis in the Indian sample (z = −3.67). However, inspection of the frequencies showed that 50% of the Indian participants recognized the symptoms represented in the vignette as a mental disorder and there was variation in the recognition scores that would allow for valid correlations, which does not support a flooring effect.

Next we tested Hypotheses 2, 3, 5, 6, and 8—associations between variables in the MHL model (see Figure 1). First, we conducted *t*-tests to examine cultural differences in predictor and outcome variables. There were no significant cultural differences in collectivism and social causal beliefs (Table 4). However, Indians held significantly more positive lay help-seeking beliefs, while significantly more negative professional help-seeking beliefs (Table 4). Also, the European Americans were significantly better at recognizing the mental disorders displayed in the vignettes than their Indian counterparts; this pattern held across all three mental disorders (Table 4).

**Table 4:** **Demographic variables—means, standard deviations and difference tests**.

	**Culture**	***M***	***SD***	***t***	**df**	***p***
Collectivism	European American	25.90	5.80	−5.43	206	0.47
	Indian	30.20	5.60			
Professional help-seeking beliefs	European American	18.76	7,12	−3.63	206	0.001
	Indian	15.28	6,67			
Lay help-seeking beliefs	European American	61.47	19,45	4.42	206	0.001
	Indian	73.23	18,83			
Social causal beliefs	European American	15.78	4,47	1.14	206	0.26
	Indian	15.04	4,84			
Recognition	European American	4.18	1.44	14.56	204	0.001
	Indian	1.32	1.34			
Depression	European American	1.80	0.56	11.42	206	0.001
	Indian	0.66	0.86			
Schizophrenia	European American	1.44	0.71	12.04	206	0.001
	Indian	0.38	0.53			
GAD	European American	0.93	0.69	7.59	204	0.001
	Indian	0.29	0.50			

Before commencing model testing, we created item parcels to represent the observed variables. We created parcels by conducting exploratory factor analyses on the items of the measures; these items were then ranked according to the size of their factor loadings (Russell et al., 1998). The highest loading items were paired with the lowest loading items and assigned to a parcel, so that parcels reflected the latent variables equally. We created two parcels for all latent variables.

We used multi-group structural equation modeling to test the hypothesized moderated-mediation model (see Figure 1). The model held a good fit when allowed to vary freely across groups (see Model 1 in Table 5). Inspection of the regression weights showed some non-significant loadings in both cultural groups (*p*s > 0.05). Thus we tested further models removing any paths between latent variables that were non-significant in both cultures, until all remaining paths were significant in either cultural group. See Table 5 for order of path removal. The final model holds an optimal model fit (see Model 3 in Table 5).

**Table 5:** **Comparison of mental health literacy models—removing non-significant paths**.

	**RMSEA**										
	**χ^2^**	**df**	***p***	**CFI**		**LB**	**HB**	**SRMR**	**Δχ**^**2**^	**df**	***p***
Model 1	83.67	52	0.004	0.96	0.05	0.03	0.08	0.07			
Model 2: removed *Social Causal beliefs → Professional help-seeking beliefs*	83.86	54	0.006	0.96	0.05	0.03	0.07	0.07	0.19	2	0.91
Model 3: removed *Collectivism → Professional help-seeking beliefs*	85.75	56	0.006	0.96	0.05	0.03	0.07	0.06	1.89	2	0.39

**Figure 8:**
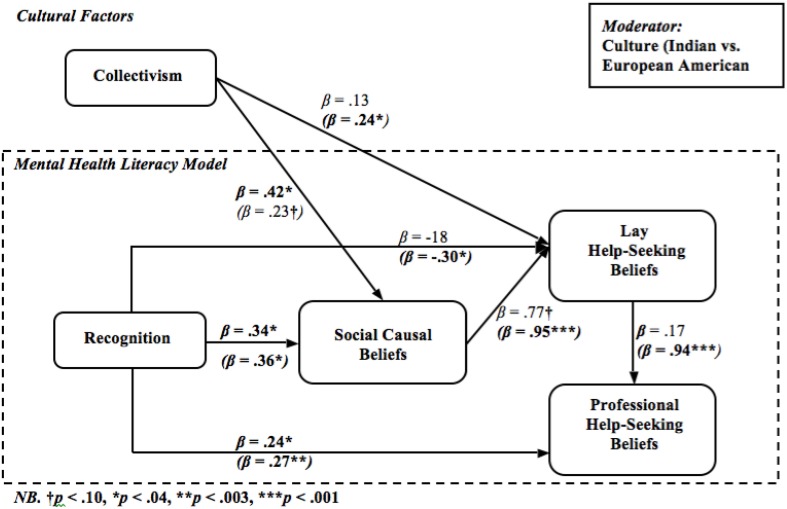
**Final mental health literacy model: standardized beta values—European American (***Indian***)**. Significant loadings are bolded.

Next, the paths in the final model were examined. *Hypothesis 2* was refuted, as collectivism did not significantly predict professional help-seeking beliefs in either cultural group (Figure 8). *Hypothesis 3* was partially supported as collectivism significantly, positively predicted lay help-seeking beliefs in the Indian sample (Figure 8). This indicates that Indian participants who reported greater collectivism were also more likely to positively endorse lay help-seeking beliefs. The association was significantly moderated by culture as the association was non-significant in the European American sample, lending some insight into the research question—whether culture will moderate the MHL model.

All aspects of *Hypothesis 5* were either fully or partially confirmed (Figure 8). First, recognition significantly positively predicted professional help-seeking beliefs in both cultures—supporting to *H5(i)* – and also significantly negatively predicted lay help-seeking beliefs in the Indian sample—lending partial support to *H5(ii)*. This indicates that, on the one hand, both European American and Indians who were better at recognizing mental disorders were more likely to endorse seeking professional help and, on the other hand, Indians who demonstrated better recognition were less likely to endorse seeking lay help. Thus cultural group significantly moderated the association between recognition and lay help-seeking beliefs, lending further support to the research question—how culture will moderate the MHL model.

Next we examined *Hypothesis 6*—associations of social causal beliefs within the MHL model (Figure 8). First, lending partial support to *H6(a)(i)* social causal beliefs were significantly positively associated with lay help-seeking beliefs in the Indian sample, while the association trended toward significance in the European American sample. This indicates that Indian participants who reported greater agreement with social causes were also more likely to endorse seeking lay help. Further, this lends greater insight into the *RQ*—how the MHL model is moderated by culture. Third, *H6(a)(ii)* was refuted as the association between social causal beliefs and professional help-seeking beliefs was non-significant in both cultural groups (*p* > 0.05). Finally, *H6(b)* related to biological causal beliefs and because we did not find cross-cultural validity for the biological causal beliefs subscale we were not able to examine this hypothesis.

Next, lending support to *H8(i)*, collectivism significantly positively predicted social causal beliefs in the European American sample, while the association trended toward significance in the Indian sample. This indicates that European American participants who reported greater collectivism were more likely to believe symptoms described in the vignette were due to social causes. Finally, *H8(ii)* related to biological causal beliefs, thus we were not able to examine these hypotheses.

Moreover, the association between lay and professional help-seeking beliefs was significant and positive in the Indian sample but non-significant in the European American sample (Figure 8). A chi difference test confirmed cultural moderation (Table 6). In the Indian sample greater endorsement of seeking lay help predicted greater endorsement to seek help from a professional, whereas in the European American sample the relationship between these two variables was orthogonal. These finding demonstrate how the MHL model was moderated by culture, lending further insight into our research question.

**Table 6:** **Cultural equivalence of model pathways**.

			**χ^2^**	**df**	***p***
Social Causal beliefs	←	Collectivism	0.17	1	.68
Social Causal beliefs	←	Recognition	10.83	1	0.001
Lay help-seeking beliefs	←	Social Causal beliefs	0.16	1	0.69
Lay help-seeking beliefs	←	Recognition	0.33	1	0.57
Professional help-seeking beliefs	←	Recognition	0.00	1	0.98
Professional help-seeking beliefs	←	Lay help-seeking beliefs	21.89	1	0.02
Lay help-seeking beliefs	←	Collectivism	0.69	1	0.41

### Hypotheses 4, 7, and 9: Indirect effects

In the final step we tested the indirect effects within the MHL literacy model—examining Hypotheses 4 and 7. The indirect effects were tested via a bootstrapping procedure (Shrout and Bolger, 2002) that examined the 95% bias-corrected confidence intervals (CI) from 1000 bootstrap samples.

Lending partial support to *Hypotheses 4(a)* and *9* we found significant indirect effects between collectivism as well as social causal beliefs and professional help-seeking beliefs through lay help-seeking beliefs in the Indian sample (Table 7). This indicates that Indian participants who reported greater collectivism and greater endorsement of social causal beliefs were more likely to endorse seeking lay help and were in turn more likely to hold positive beliefs about seeking help from a professional. These indirect effects were however non-significant in the European American sample.

**Table 7:** **Indirect effects of variables within the MHL model**.

			**European American**				**Indian**			
**IV**	**Mediator**	**DV**	**β**	**LB**	**HB**	***p***	**β**	**LB**	**HB**	***p***
Recognition	Social causal beliefs	Lay help-seeking beliefs	6.39	0.00	0.58	0.03	9.47	0.74	26.21	0.07
Collectivism	Social causal beliefs	Lay help-seeking beliefs	0.78	0.06	6.02	0.007	0.77	0.00	2.67	0.15
Recognition	Lay help-seeking beliefs	Professional help-seeking beliefs	−0.19	−1.58	0.15	0.19	−0.43	−3.80	2.72	0.83
Collectivism	Lay help-seeking beliefs	Professional help-seeking beliefs	0.10	−0.36	0.07	0.29	0.65	0.18	1.17	0.004
Social causal beliefs	Lay help-seeking beliefs	Professional help-seeking beliefs	0.38	−0.18	3.37	0.13	2.16	4.91	1.11	0.002

*Hypothesis 4(b)* was refuted, as the indirect effect between recognition and professional help-seeking beliefs via lay help-seeking beliefs was non-significant in both cultural groups (Table 7).

*Hypothesis 7(a)(i)* was partially supported—recognition was indirectly associated with greater lay help-seeking beliefs via greater social causal beliefs in the European American sample and the association trended toward significance in the Indian sample (Table 7). This indicates that participants who were better at recognizing symptoms of mental illness were more likely to believe that these symptoms were caused by social circumstances, which in turn predicted greater endorsement of lay help-seeking. Further, *Hypothesis 7(b) (i)* was partially confirmed as collectivism was indirectly associated with lay help-seeking beliefs through social causal beliefs in the European American sample, while this was not the case in the Indian sample (Table 7). This indicates that European American participants who reported greater collectivism were more likely to believe symptoms of mental illness had social causes, which in turn was associated with greater endorsement of seeking lay help. Furthermore, Moreover, *Hypothesis 7(a) (ii)* and *7(b) (ii)* were refuted, as the association between social causal beliefs and professional help-seeking beliefs was non-significant in both cultural groups (*p* > 0.05).

Finally, the findings of associations between variables and indirect effects within the MHL model give further insight into our *RQ*, confirming that culture acts as a moderator to the MHL model.

## Discussion

The present findings showed strong associations between the elements—recognition, causal, lay and professional help-seeking beliefs—of the proposed MHL model (Jorm et al., 1997a,b,c; Jorm, 2000, 2011), lending strong empirical support to the model. However, a cultural difference in the MHL model was evident. On the one hand, we found that lay help-seeking beliefs were associated with all other aspects of the MHL model in the Indian sample, whereas in the European American sample, lay help-seeking beliefs were not a significant part of the MHL model. On the other hand, collectivism was significantly associated with social causal beliefs in the European American sample and with lay help-seeking beliefs in the Indian sample.

### Causal beliefs, lay help-seeking beliefs and professional help-seeking beliefs measures

For all three of our MHL measures, we retained items that held cross-cultural invariance. In respect to the causal beliefs measure, the items relating to biological causes did not hold cross-cultural invariance (*hormonal imbalance* and *brain damage*). Jorm (2011) proposed that greater recognition of mental illness would activate a particular schema that outlines the type of action to take. However, as the Indian sample showed lower recognition and therefore knowledge of mental disorders, it is likely that they held different schemas about mental illness, therefore making biological causal beliefs irrelevant. Further, the items retained for social causal beliefs appear to be broader (e.g., *traumatic event* and *stress*), while items that did not hold equivalence may have been too situation- or person-specific (e.g., *problems at work* or *relationship problems*).

In relation to the professional help-seeking beliefs measure, the retained items reflect a biological model of mental illness (e.g., *take medication* and *go to psychiatric clinic*). This may be due to perceived accessibility and availability of mental health facilities across cultures. Facilities for mental health issues are scarce in India (World Health Organisation, 2011a,b), and even basic satisfactory primary health care is lacking in many rural areas (Khandelwal et al., 2004). Therefore, more specific professional psychological help—like psychologists or psychiatrists—may not have been recognized or perceived as relevant to mental health issues in the Indian sample.

Furthermore, most items of the lay help-seeking beliefs measure were retained, which is in line with previous findings that across cultures individuals with mental health issues draw support from a wide range of informal sources (Cooper-Patrick et al., 1997; Van Hook, 1999; Chadda et al., 2001). This further indicates that there is less cross-cultural variability in potential sources of lay help, although perceived helpfulness of these lay sources may still vary.

### Mental disorders

On the one hand, we aimed to establish cross-cultural validity for the novel MHL measures, and on the other hand we intended to examine three of the most prevalent mental disorders worldwide—depression, schizophrenia, and GAD. Unfortunately, the GAD data did not yield strong results and we were not able to use the GAD data in our analyses. This may be because compared to GAD, depression and schizophrenia receive greater attention in books, TV shows, movies, and awareness campaigns (Tartakovsky, 2011). Although the media often misrepresents mental disorders (Tartakovsky, 2011), greater media attention would also indicate greater awareness of these disorders. Indeed, our results showed that compared to GAD, both the Indian and European American sample had greater awareness of the primary symptoms of depression and schizophrenia than GAD and were more likely to recognize depression and schizophrenia as mental disorders.

### MHL model

After establishing validity across cultures and mental disorders of the developed MHL measures, we examined the MHL model itself. Overall, we found strong quantitative support for the MHL model. We found that recognition of symptoms of mental illness was the strongest predictor of all other aspects of the MHL model. In both cultures greater recognition was associated with more positive professional help-seeking beliefs, while in the Indian sample it was also positively associated with lay help-seeking beliefs. This gives support to the literature that stresses the link between knowing about symptoms of mental illness and endorsement of seeking appropriate help for these symptoms (Jorm et al., 1997b; Lauber et al., 2003; Wright et al., 2007).

Our findings showed an overwhelming cultural difference in the MHL model. The main cultural difference was that in the Indian sample, lay help-seeking beliefs was a major variable, while in the European American sample it did not have any significant associations with other aspects of the model. From our findings it appears that the European American sample did not view lay help as a relevant source of help in relation to mental health issues. Contrary to our findings, previous literature stressed that across cultures the public appears to hold a preference for seeking help from lay as opposed to professional sources when faced with issues of mental health (Cooper-Patrick et al., 1997; Jorm et al., 1997c; Van Hook, 1999). This indicates that European American participants either perceived lay help as not important in relation to mental health issues or that they were not able to draw on lay sources of help. On the contrary, in the Indian sample lay and professional help-seeking beliefs were highly positively related, which indicates that Indian participants believed that professional sources significantly positively complemented lay ones. Previous literature revealed that in the Indian culture the family holds the main responsibility for treatment and care for a person with mental illness (Khandelwal et al., 2004) and it is more likely that professional help is sought when the in-group endorsed this (Shankar et al., 2006). The present findings showed that Indian participants endorsed lay help to a greater extent than professional help, thus supporting previous studies in that professional help is viewed as merely an additional source of help.

Furthermore, the majority of the MHL literature has examined professional help-seeking beliefs (Tata and Leong, 1994; Kuo et al., 2007; ten Have et al., 2010), our findings underline the importance of examining lay help-seeking beliefs as well, because it appears that Indians perceive lay help-seeking as a predictor of professional help-seeking.

Moreover, we found an indirect effect between collectivism and professional help-seeking beliefs through lay help-seeking beliefs in the Indian sample, with greater collectivism being related to more positive beliefs about seeking lay help and in turn greater endorsement of seeking professional help. Interestingly, contrary to previous findings (Tata and Leong, 1994; Yeh, 2002; Kuo et al., 2007), we did not find a direct relationship between collectivism and professional help-seeking beliefs in either culture. It appears that in highly collectivist cultures, individuals are more likely to perceive seeking help from lay sources as favorable, and in turn they would positively perceive seeking help from professionals.

Further, contrary to previous research (Sheikh and Furnham, 2000; Chen and Mak, 2008), endorsement of social causes of mental illness was not related to professional help-seeking beliefs. This may be because we were only able to examine social causes, whereas biological causes may have been more relevant to professional help-seeking beliefs. However, lending some support to previous findings (Chen and Mak, 2008; Sheikh and Furnham, 2000), we did find that social causal beliefs were indirectly related to professional help-seeking beliefs through lay help-seeking beliefs in the Indian sample. Furthermore, as predicted, greater endorsement of social causes was related to more positive lay help-seeking beliefs in the Indian sample. That is, individuals who believed that mental illness was due to social causes were also more likely to endorse reaching out to the social environment to manage these symptoms.

Finally, in the European American sample greater collectivism was associated with greater endorsement of social causal beliefs. The literature suggested that collectivists would be more likely to attribute causes of mental illness to the community (Narikiyo and Kameoka, 1992; Dietrich et al., 2003; Speller, 2005). One reason that we only found this significant association in the European American sample may be because more collectivist cultures already heavily rely on social explanations for mental illness (Shankar et al., 2006; Penny et al., 2009). Thus, social causes of mental disorders may be the baseline explanation in Indian cultures, whereas in the European American sample it may be perceived as one of several explanations.

### Strengths, limitations, and future directions

One of the main strengths of the present study was that it adopted a quantitative approach to examine the MHL model. This is in contrast to many studies that either employed a qualitative approach (Jorm et al., 1997a,b,c; Jorm, 2000, 2011), or only studied particular aspects of MHL quantitatively (Atkinson et al., 1991; Tata and Leong, 1994; Yeh, 2002; Kuo et al., 2007). Neither approach facilitates the examination of the elements of MHL with each other, nor does it allow the study of MHL in relation to other variables. In the present study we developed MHL measures that held cross-cultural validity, which enabled us to attend to these shortcomings, further identify cultural differences in MHL, and identify collectivism as a predictor of MHL.

A further strength of the present study was that we examined professional as well as lay help-seeking beliefs. Most studies investigating MHL have solely focused on professional help-seeking (Tata and Leong, 1994; Kuo et al., 2007; ten Have et al., 2010), despite the public's preference and wide use of lay sources (Cooper-Patrick et al., 1997; Jorm et al., 1997c; Van Hook, 1999; Chadda et al., 2001). The present study rectified this shortcoming and found that lay help-seeking beliefs were indeed a crucial aspect of MHL in the Indian sample. Further, the literature showed that some cultures—including India—rely heavily on traditional and spiritual healers (Khandelwal et al., 2004). Because, alternative medicine and spiritual leaders are core sources of help in some cultures, future research should examine whether they should be incorporated into the MHL model.

The present approach necessarily had limitations. First, presentation of the symptoms of mental illness may vary between cultures (Williams and Healy, 2001; Bhugra, 2006), engendering the possibility that Indians were lower in recognition because these symptoms were irrelevant to their culture. It is important to note, however, that although interpretations of mental illness can vary between cultures, the core symptoms of mental illness remain universal (Bhugra, 2006; Williams and Healy, 2001), and the vignettes used in the current study focused on the core symptoms.

Another limitation stems from the use of the expectation-maximization method to infer missing values, and therefore findings need to be interpreted with caution. However, the alleged shortcomings of the expectation-maximization method—e.g., multiple modes, saddlepoints, ridges—should be interpreted as inherent features of the algorithm, because expectation-maximization fares well in comparison to other computational methods (Schafer, 1997).

A further limitation stems from the recognition scale we used, which was a reflection of knowledge of mental disorders. Future research may want to use a more detailed knowledge scale to be able to discriminate between categorizing someone as ill as opposed to knowing what is going on with the person.

In conclusion, our study revealed significant cultural differences in knowledge as well as beliefs about causes and help-seeking for mental illness. This underlines the importance of understanding beliefs of mental illness in different cultures in order to develop more effective, approachable, and culturally sensitive mental health care systems. In doing so individuals like the Rwandan man, who was puzzled by the cultural differences in mental health care, do not need to wonder what “bizarre things” mental health workers are providing.

### Conflict of interest statement

The authors declare that the research was conducted in the absence of any commercial or financial relationships that could be construed as a potential conflict of interest.

## Footnotes

^1^First we ran exploratory factor analyses on the newly developed scales, however the results were random and uninterpretable. These findings were likely driven by poor items, which were revealed by subsequent analyses.
