# Supplementary material for: Mental health literacy: a cross-cultural approach to knowledge and beliefs about depression, schizophrenia and generalized anxiety disorder
Source: Front Psychol. 2015 Sep 8;6:1272. doi: 10.3389/fpsyg.2015.01272 (PMC4561812; doi:10.3389/fpsyg.2015.01272)
Supplement: Supplementary file 1 [file Table1.DOCX]

## Supplementary File: Tables

Table S1. Demographic Variables – Means, standard deviations and chi-square and *t*-test tests.

|  | Culture | M | *SD* | *t* | df | *p* |
| --- | --- | --- | --- | --- | --- | --- |
| Age | European American | 34.78 | 13.13 | 2.32 | 206 | .007 |
|  | Indian | 30.90 | 10.78 |  |  |  |
|  |  | Frequencies | | *X^2^* | df | *p* |
| Gender |  | European American | Indian |  |  |  |
|  | Female | 67 | 41 | 14.08 | 1 | .001 |
|  | Male | 41 | 64 |  |  |  |
| Education | Lower | 54 | 19 | 23.04 | 1 | .001 |
|  | Higher | 51 | 80 |  |  |  |
| Religion | Christian | 19 | 49 | 305.55 | 6 | .001 |
|  | Muslim | 15 | 0 |  |  |  |
|  | Jewish | 0 | 2 |  |  |  |
|  | Hindu | 89 | 1 |  |  |  |
|  | Buddhist | 0 | 2 |  |  |  |
|  | Non-religious | 1 | 35 |  |  |  |

Table S2. Scale items of the Social Causal beliefs, Professional help-seeking beliefs and Lay help-seeking beliefs measures (retained scale items shaded in grey).

| Scale Items | Causal beliefs | Lay help-seeking beliefs | Professional help-seeking beliefs |
| --- | --- | --- | --- |
| 1 | problems with their family | talk to their children | see a psychologist |
| 2 | brain damage | see a spiritual leader (e.g. priest, imam) | see a GP / doctor |
| 3 | hormonal imbalance | get some fresh air | see a psychiatrist |
| 4 | problems at work | take some vitamins | go for counselling and/or therapy |
| 5 | loss of a loved one | talk to their spouse | go to a psychiatric clinic |
| 6 | experienced a traumatic event | go on a holiday | take medication (e.g. antidepressants / antibiotics) |
| 7 | been experiencing too much stress | talk to a teacher / professor / lecturer | call a telephone helpline |
| 8 | relationship problems | talk to friends | - |
| 9 | - | talk to a colleague | - |
| 10 | - | read about mental illness (in a book or on the internet) | - |
| 11 | - | talk to their parents | - |

Table S3. Scale means, standard deviations and reliability coefficients.

| European American /  (Indian) | Causal beliefs | | | Professional help-seeking beliefs | | | Lay help-seeking beliefs | | |
| --- | --- | --- | --- | --- | --- | --- | --- | --- | --- |
|  | Depression | Schizophrenia | GAD | Depression | Schizophrenia | GAD | Depression | Schizophrenia | GAD |
| α |  |  |  |  |  |  |  |  |  |
| All items | .88  (.77) | .84  (.81) | .72  (.71) | .88  (.87) | .80  (.88) | .83  (.83) | .89  (.88) | .91  (.88) | .87  (.86) |
| Final items | .81  (.60) | .74  (.76) | .64  (.45) | .73  (.63) | .60  (.59) | .70  (.64) | .86  (.86) | .91  (.85) | .86  (.84) |
| Mean |  |  |  |  |  |  |  |  |  |
| All items | 20.90 | 20.95 | 22.12 | 37.04 | 43.95 | 39.76 | 15.84 | 15.15 | 16.29 |
|  | (19.64) | (25.00) | (22.53) | (32.82) | (34.63) | (34.78) | (19.47) | (20.17) | (20.35) |
| Final items | 6.76 | 9.01 | 7.60 | 33.45 | 44.30 | 36.67 | 7.84 | 7.43 | 8.38 |
|  | (7.45) | (7.63) | (7.82) | (30.00) | (34.45) | (31.67) | (9.06) | (9.70) | (9.54) |
| SD |  |  |  |  |  |  |  |  |  |
| All items | 5.65 | 5.78 | 5.26 | 11.31 | 12.90 | 10.37 | 7.42 | 6.88 | 6.52 |
|  | 6.09 | 6.77 | 4.88 | (11.47) | (11.44) | (11.09) | (8.28) | (8.31) | (7.82) |
| Final items | 2.49 | 3.04 | 2.29 | 10.30 | 13.12 | 9.60 | 3.87 | 3.67 | 3.57 |
|  | (2.55) | (2.85) | (2.38) | (10.37) | (11.07) | (10.00) | (3.72) | (3.58) | (3.75) |

Table S4. Correlations for the causal beliefs, professional help-seeking beliefs and lay help-seeking beliefs measures by sample (European American under axis, Indian over axis shaded in grey).

|  | **Causal beliefs - Depression** | | | | | | | | | | | | | | **Causal beliefs - Schizophrenia** | | | | | | | | | | | | | | | | | | |  |
| --- | --- | --- | --- | --- | --- | --- | --- | --- | --- | --- | --- | --- | --- | --- | --- | --- | --- | --- | --- | --- | --- | --- | --- | --- | --- | --- | --- | --- | --- | --- | --- | --- | --- | --- |
|  | Item 1 | Item 2 | | Item 3 | | Item 4 | | Item 5 | | Item 6 | | Item 7 | | Item 8 |  | | Item 1 | | Item 2 | | | Item 3 | | Item 4 | | Item 5 | | Item 6 | | Item 7 | | Item 8 | |  |
| 1 |  | .54** | | .51** | | .58** | | .59** | | .62** | | .42** | | .24* | 1 | |  | | .34** | | | .50** | | .72** | | .64** | | .70** | | .34** | | .03 | |  |
| 2 | .38** |  | | .69** | | .61** | | .81** | | .70** | | .48** | | .35** | 2 | | .36** | |  | | | .63** | | .32** | | .55** | | .42** | | .30** | | .16 | |  |
| 3 | .18 | .43** | |  | | .60** | | .64** | | .54** | | .36** | | .14 | 3 | | .52** | | .64** | | |  | | .49** | | .58** | | .51** | | .30** | | .01 | |  |
| 4 | .23* | .47** | | .50** | |  | | .69** | | .66** | | .31** | | .14 | 4 | | .28** | | .38** | | | .39** | |  | | .63** | | .66** | | .27** | | .02 | |  |
| 5 | .45** | .62** | | .21* | | .41** | |  | | .70** | | .4** | | .35** | 5 | | .35** | | .55** | | | .38** | | .24* | |  | | .61** | | .41** | | .19 | |  |
| 6 | .60** | .57** | | .38** | | .37** | | .48** | |  | | .43** | | .31** | 6 | | .56** | | .35** | | | .49** | | .32** | | .33** | |  | | .36** | | -.02 | |  |
| 7 | .05 | .24* | | .17 | | .15 | | .23* | | .05 | |  | | .33** | 7 | | .30** | | .15 | | | .24* | | .27** | | .19 | | .19 | |  | | .28** | |  |
| 8 | .19 | .12 | | -.02 | | .07 | | .06 | | .13 | | .49** | |  | 8 | | .40** | | .30** | | | .27** | | .12 | | .23* | | .27** | | .51** | |  | |  |
| *** p > .001, ** *p* > .01, **p* > .05, †*p* > .1 | | | | | | | | | | | | | | | | | | | | | | | | | | | | | | | | | |  |
|  | **Causal beliefs - GAD** | | | | | | | | | | | | | | **Professional help-seeking beliefs - Depression** | | | | | | | | | | | | | | | | | | |  |
|  | Item 1 | Item 2 | | Item 3 | | Item 4 | | Item 5 | | Item 6 | | Item 7 | | Item 8 |  | | | Item 1 | | | Item 2 | Item 3 | | Item 4 | | Item 5 | | Item 6 | | | Item 7 | | |  |
| 1 |  | .22* | | .32** | | .21* | | .25** | | .51** | | -.01 | | .09 | 1 | | |  | | | .24* | .29** | | .29** | | .29** | | .29** | | | .19† | | |  |
| 2 | .08 |  | | .64** | | .02 | | .64** | | .34** | | .14 | | .08 | 2 | | | .45*** | | |  | .61*** | | .58*** | | .60*** | | .55*** | | | .55*** | | |  |
| 3 | .12 | .51** | |  | | .16 | | .51** | | .44** | | .08 | | .00 | 3 | | | .37*** | | | .61*** |  | | .68*** | | .68*** | | .52*** | | | .63*** | | |  |
| 4 | .33** | .12 | | .24* | |  | | .28** | | .36** | | .08 | | .24* | 4 | | | .41*** | | | .50*** | .57*** | |  | | .68*** | | .50*** | | | .71*** | | |  |
| 5 | .23* | .41** | | .30** | | .07 | |  | | .42** | | .11 | | .17 | 5 | | | .44*** | | | .56*** | .61*** | | .55*** | |  | | .48*** | | | .68*** | | |  |
| 6 | .50** | .11 | | .26** | | .24* | | .298** | |  | | .09 | | .20* | 6 | | | .38*** | | | .50*** | .51*** | | .47*** | | .68*** | |  | | | .61*** | | |  |
| 7 | .15 | .15 | | .10 | | .19 | | .043 | | .11 | |  | | .41** | 7 | | | .19 | | | .55** | .63** | | .71** | | .68** | | .61** | | |  | | |  |
| 8 | .33** | .14 | | .36** | | .15 | | .219* | | .38** | | .30** | |  |  | | |  | | |  |  | |  | |  | |  | | |  | | |  |
| *** p > .001, ** *p* > .01, **p* > .05, †*p* > .1 | | | | | | | | | | | | | | | | | | | | | | | | | | | | | | | | | |  |
| **Professional help-seeking beliefs – Schizophrenia** | | | | | | | | | | | | | | | **Professional help-seeking beliefs - GAD** | | | | | | | | | | | | | | | | | | |  |
|  | Item 1 | | Item 2 | | Item 3 | | Item 4 | | Item 5 | | Item 6 | | Item 7 | |  | Item 1 | | | | Item 2 | | | Item 3 | | Item 4 | | Item 5 | | Item 6 | | | | Item 7 | |
| 1 |  | | .27** | | .16 | | .27** | | .34*** | | .40*** | | .26** | | 1 |  | | | | .20* | | | .11 | | .27** | | .22* | | .41*** | | | | -.005 | |
| 2 | .19* | |  | | .56*** | | .62*** | | .55*** | | .53*** | | .49*** | | 2 | .30*** | | | |  | | | .56*** | | .39*** | | .48*** | | .30** | | | | .46*** | |
| 3 | .20* | | .64*** | |  | | .58*** | | .57*** | | .56*** | | .61*** | | 3 | .34*** | | | | .48*** | | |  | | .58*** | | .58*** | | .26** | | | | .50*** | |
| 4 | .20* | | .33*** | | .53*** | |  | | .72*** | | .55*** | | .59*** | | 4 | .36*** | | | | .31*** | | | .41*** | |  | | .54*** | | .45*** | | | | .54*** | |
| 5 | .11 | | .52*** | | .71*** | | .46*** | |  | | .69*** | | .73*** | | 5 | .15 | | | | .44*** | | | .55*** | | .48*** | |  | | .50*** | | | | .63*** | |
| 6 | .22* | | .37*** | | .56*** | | .28** | | .45*** | |  | | .65*** | | 6 | .29** | | | | .44*** | | | .43*** | | .32*** | | .51*** | |  | | | | .53*** | |
| 7 | .21* | | .23* | | .47*** | | .44*** | | .58*** | | .52*** | |  | | 7 | .18† | | | | .49*** | | | .47*** | | .56*** | | .76*** | | .45*** | | | |  | |
| *** p > .001, ** *p* > .01, **p* > .05, †*p* > .1 | | | | | | | | | | | | | | | | | | | | | | | | | | | | | | | | | |  |

Table S4. Correlations for the causal beliefs, professional help-seeking beliefs and lay help-seeking beliefs measures by sample (European American under axis, Indian over axis shaded in grey) Continued.

|  | **Lay help-seeking beliefs - Depression** | | | | | | | | | | |
| --- | --- | --- | --- | --- | --- | --- | --- | --- | --- | --- | --- |
|  | Item 1 | Item 2 | Item 3 | Item 4 | Item 5 | Item 6 | Item 7 | Item 8 | Item 9 | Item 10 | Item 11 |
| 1 |  | .54** | .08 | .21* | .34** | .68** | .59** | .35** | .60** | .56** | .61** |
| 2 | .53*** |  | .34** | .25* | .39** | .59** | .54** | .31** | .56** | .50** | .46** |
| 3 | .38*** | .32*** |  | .14 | .25* | .10 | .19 | .26* | .22* | .27** | .08 |
| 4 | .41*** | .34*** | .24** |  | .36** | .31** | .11 | .17 | .23* | .14 | .26** |
| 5 | .45*** | .48*** | .08 | .43*** |  | .51** | .40** | .28** | .45** | .44** | .63** |
| 6 | .43*** | .39*** | .30** | .45*** | .62*** |  | .61** | .36** | .63** | .52** | .61** |
| 7 | .76*** | .51*** | .31*** | .34*** | .45*** | .45*** |  | .57** | .66** | .58** | .56** |
| 8 | .45*** | .36*** | .27** | .37*** | .56*** | .45*** | .40*** |  | .54** | .49** | .34** |
| 9 | .51*** | .45*** | .45*** | .45*** | .35*** | .49*** | .45*** | .44*** |  | .70** | .55** |
| 10 | .76*** | .56*** | .34*** | .37*** | .54*** | .45*** | .75*** | .51*** | .42*** |  | .57** |
| 11 | .58*** | .58*** | .34*** | .40*** | .48*** | .30** | .49*** | .45*** | .52*** | .56*** |  |
| *** p > .001, ** *p* > .01, **p* > .05, †*p* > .1 | | | | | | | | | | | |
|  | **Lay help-seeking beliefs - Schizophrenia** | | | | | | | | | | |
|  | Item 1 | Item 2 | Item 3 | Item 4 | Item 5 | Item 6 | Item 7 | Item 8 | Item 9 | Item 10 | Item 11 |
| 1 |  | .47*** | .13 | .23* | .63*** | .45*** | .65*** | .64*** | .38*** | .47*** | .39*** |
| 2 | .42*** |  | .11 | .20* | .52*** | .56*** | .58*** | .36*** | .32*** | .32*** | .30*** |
| 3 | .42*** | .34*** |  | .22* | .39*** | .35*** | .24* | .12 | .18 | .28*** | .34** |
| 4 | .48*** | .37*** | .23* |  | .30** | .40*** | .26** | .15 | .25** | .28** | .27** |
| 5 | .26** | .49*** | .17 | .24** |  | .61*** | .58*** | .53*** | .34*** | .47*** | .46*** |
| 6 | .35*** | .56*** | .32*** | .25** | .70*** |  | .57*** | .51*** | .42*** | .46*** | .49*** |
| 7 | .71*** | .48*** | .29** | .47*** | .37*** | .48*** |  | .43*** | .44*** | .43*** | .39*** |
| 8 | .44*** | .61*** | .34*** | .36*** | .55*** | .69*** | .49*** |  | .42*** | .53*** | .54*** |
| 9 | .55*** | .61*** | .36*** | .43*** | .42*** | .55*** | .53*** | .56*** |  | .47*** | .27** |
| 10 | .67*** | .567*** | .43*** | .46*** | .36*** | .52*** | .61*** | .59*** | .62*** |  | .75*** |
| 11 | .67*** | .59*** | .46*** | .45*** | .27** | .53*** | .52*** | .57*** | .70*** | .75*** |  |
| *** p > .001, ** *p* > .01, **p* > .05, †*p* > .1 | | | | | | | | | | | |

Table S4. Correlations for the causal beliefs, professional help-seeking beliefs and lay help-seeking beliefs measures by sample (European American under axis, Indian over axis shaded in grey) Continued.

|  | **Lay help-seeking beliefs - GAD** | | | | | | | | | | |
| --- | --- | --- | --- | --- | --- | --- | --- | --- | --- | --- | --- |
|  | Item 1 | Item 2 | Item 3 | Item 4 | Item 5 | Item 6 | Item 7 | Item 8 | Item 9 | Item 10 | Item 11 |
| 1 |  | .50*** | .19 | .36*** | .62*** | .41*** | .47*** | .44*** | .30** | .49**** | .48**** |
| 2 | .34*** |  | .33*** | .21* | .49*** | .38*** | .49*** | .37*** | .25** | .54*** | .17 |
| 3 | .36*** | .31*** |  | .14 | .19 | .27** | .09 | .14 | .22* | .23* | .20* |
| 4 | .46*** | .45*** | .32*** |  | .35*** | .62*** | .31** | .38*** | .51*** | .36*** | .36*** |
| 5 | .13 | .41*** | .13 | .28*** |  | .60*** | .36*** | .50*** | .30** | .54*** | .49*** |
| 6 | .23* | .37*** | .27*** | .37*** | .75*** |  | .40*** | .45*** | .50*** | .41*** | .42*** |
| 7 | .54*** | .42*** | .26*** | .33*** | .44*** | .39*** |  | .39*** | .36*** | .52*** | .44*** |
| 8 | .15 | .42*** | .24** | .27*** | .70*** | .68*** | .42*** |  | .36*** | .45*** | .37*** |
| 9 | .41*** | .30*** | .35*** | .49*** | .21* | .42*** | .22* | .30*** |  | .31** | .43*** |
| 10 | .61*** | .44*** | .25** | .36*** | .29** | .42*** | .52*** | .31*** | .35*** |  | .56*** |
| 11 | .52*** | .54*** | .36*** | .55*** | .23* | .34*** | .30*** | .37** | .63*** | .33*** |  |
| *** p > .001, ** *p* > .01, **p* > .05, †*p* > .1 | | | | | | | | | | | |

Table S5. Factor loadings and chi square test of invariance of the initial professional help-seeking beliefs measure (see Figure X; significant factor loadings are bolded).

|  | Depression | | | | | Schizophrenia | | | | | GAD | | | | |
| --- | --- | --- | --- | --- | --- | --- | --- | --- | --- | --- | --- | --- | --- | --- | --- |
|  | European American | Indian | X^2^ | df | *p* | European American | Indian | X^2^ | df | *p* | European American | Indian | X^2^ | df | *p* |
| See psychologist | **0.84** | **0.82** | 3.76 | 1 | .05 | **0.60** | **0.81** | 9.03 | 1 | .003 | **0.84** | **0.77** | 3.65 | 1 | .06 |
| See GP | **0.75** | **0.66** | .10 | 1 | .75 | **0.61** | **0.78** | 3.81 | 1 | .05 | **0.59** | **0.55** | .17 | 1 | .68 |
| See psychiatrist | **0.88** | **0.82** | 1.28 | 1 | .26 | **0.80** | **0.87** | 4.53 | 1 | .03 | **0.87** | **0.80** | 3.36 | 1 | .07 |
| Go to counselling / therapy | **0.67** | **0.83** | 6.07 | 1 | .01 | **0.58** | **0.79** | 5.96 | 1 | .02 | **0.67** | **0.74** | 4.94 | 1 | .03 |
| Go to psychiatric clinic | **0.71** | **0.81** | .65 | 1 | .42 | **0.90** | **0.71** | .16 | 1 | .69 | **0.64** | **0.71** | 1.19 | 1 | .28 |
| Take medication | **0.67** | **0.72** | .02 | 1 | .89 | **0.66** | **0.68** | .73 | 1 | .39 | **0.58** | **0.66** | .81 | 1 | .37 |
| Call telephone helpline | **0.55** | **0.33** | 2.14 | 1 | .14 | **0.24** | **0.37** | .74 | 1 | .39 | **0.35** | **0.24** | .33 | 1 | .57 |

Table S6. Factor loadings and chi square test of invariance of the initial lay help-seeking beliefs measure (see Figure X; significant factor loadings are bolded).

|  | Depression | | | | | Schizophrenia | | | | | GAD | | | | |
| --- | --- | --- | --- | --- | --- | --- | --- | --- | --- | --- | --- | --- | --- | --- | --- |
|  | European American | Indian | X^2^ | df | *p* | European American | Indian | X^2^ | df | *p* | European American | Indian | X^2^ | df | *p* |
| Talk to their children | **.67** | **.69** | .06 | 1 | .82 | **-.72** | **.62** | 1.35 | 1 | .25 | **.66** | **.55** | .38 | 1 | .54 |
| See spiritual healer | **.43** | **.25** | 1.54 | 1 | .22 | **-.49** | **.35** | .36 | 1 | .55 | **.46** | **.28** | 1.24 | 1 | .27 |
| Fresh air | **.64** | **.73** | 1.79 | 1 | .08 | **-.52** | **.78** | 4.76 | 1 | .03 | **.53** | **.74** | 4.00 | 1 | .06 |
| Take vitamins | **.59** | **.59** | .01 | 1 | .92 | **-.69** | **.75** | .05 | 1 | .82 | **.59** | **.73** | 1.07 | 1 | .30 |
| Talk to spouse | **.81** | **.79** | .05 | 1 | .82 | **-.71** | **.74** | 2.92 | 1 | .09 | **.64** | **.59** | .03 | 1 | .86 |
| Go on holiday | **.60** | **.78** | 2.08 | 1 | .15 | **-.74** | **.70** | 3.52 | 1 | .06 | **.58** | **.66** | .64 | 1 | .42 |
| Talk to teacher / professor / lecturer | **.62** | **.56** | .02 | 1 | .90 | **-.78** | **.54** | 1.79 | 1 | .18 | **.59** | **.56** | .17 | 1 | .68 |
| Talk to friends | **.85** | **.83** | .29 | 1 | .59 | **-.84** | **.68** | 2.78 | 1 | .10 | **.73** | **.66** | .00 | 1 | .99 |
| Talk to colleagues | **.69** | **.75** | .82 | 1 | .37 | **-.83** | **.65** | 1.14 | 1 | .29 | **.72** | **.65** | .01 | 1 | .92 |
| Read about mental illness | **.52** | **.30** | 1.23 | 1 | .27 | **-.54** | **.38** | .32 | 1 | .58 | **.68** | **.60** | .18 | 1 | .67 |
| Talk to parents | **.85** | **.75** | .07 | 1 | .80 | **-.73** | **.74** | 1.16 | 1 | .28 | **.66** | **.70** | .34 | 1 | .56 |

Table S7. Factor loadings and chi square test of invariance of the initial causal beliefs measure (see Figure X; significant factor loadings are bolded).

|  | Depression | | | | | Schizophrenia | | | | | GAD | | | | |
| --- | --- | --- | --- | --- | --- | --- | --- | --- | --- | --- | --- | --- | --- | --- | --- |
|  | European American | Indian | X^2^ | df | *p* | European American | Indian | X^2^ | df | *p* | European American | Indian | X^2^ | df | *p* |
| Family Problems | **0.68** | **0.57** | .21 | 1 | .64 | **0.83** | **0.65** | 3.21 | 1 | .07 | **0.41** | **0.56** | 1.43 | 1 | .23 |
| Brain damage | **0.47** | **0.55** | .07 | 1 | .79 | 0.21 | **0.80** | 9.55 | 1 | .002 | **0.65** | **0.85** | .63 | 1 | .43 |
| Hormonal imbalance | **0.71** | **0.90** | .30 | 1 | .59 | 1.29 | **0.63** | 4.10 | 1 | .04 | **0.62** | **0.35** | .88 | 1 | .35 |
| Work / school / university problems | **0.76** | **0.58** | .17 | 1 | .68 | **0.80** | **0.48** | 10.21 | 1 | .001 | **0.25** | **0.38** | .81 | 1 | .37 |
| Lost a loved one | **0.90** | **0.71** | .01 | 1 | .91 | **0.80** | **0.56** | 5.23 | 1 | .02 | **0.75** | **0.48** | 2.42 | 1 | .12 |
| Traumatic event | **0.88** | **0.81** | .14 | 1 | .71 | **0.55** | **0.73** | 2.43 | 1 | .12 | **0.78** | **0.40** | 3.07 | 1 | .08 |
| Stress | **0.73** | **0.50** | .45 | 1 | .50 | **0.67** | **0.80** | .00 | 1 | .948 | **0.76** | **0.53** | .40 | 1 | .53 |
| Relationship Problems | **0.80** | **0.73** | .11 | 1 | .74 | **0.80** | **0.62** | 7.26 | 1 | .007 | **0.57** | **0.64** | .65 | 1 | .42 |
| Social <-> Biological | **0.74** | 0.26 | 7.82 | 1 | .005 | 0.34 | **0.50** | .50 | 1 | .48 | 0.23 | **0.65** | 2.60 | 1 | .11 |
