# Supplementary material for: Mental health literacy: a cross-cultural approach to knowledge and beliefs about depression, schizophrenia and generalized anxiety disorder
Source: Front Psychol. 2015 Sep 8;6:1272. doi: 10.3389/fpsyg.2015.01272 (PMC4561812; doi:10.3389/fpsyg.2015.01272)
Supplement: Supplementary file 2 [file DataSheet1.PDF]

Altweck L, Marshall TC, Ferenczi N and Lefringhausen K (2015). Mental health literacy: a cross-cultural approach to knowledge and beliefs about depression, schizophrenia and generalized anxiety disorder. ***Front. Psychol.*** **6:1272. doi: 10.3389/fpsyg.2015.01272**

Full Questionnaire

What is your age? ______________

What is your gender? Male ☐ Female ☐ Other: ___________

If English is not your first language, how well would you say do you understand and communicate in English?

Poor	Fair	Good	Excellent	Fluent	English is my first language
☐	☐	☐	☐	☐	☐

What is your highest level of <u>completed</u> education?

- ☐ Less than high school or equivalent
- ☐ High school (or equivalent) graduate
- ☐ Some university (post-secondary education, college, associate degree, technical degree)
- ☐ University graduate (College of equivalent)
- ☐ Masters degree or equivalent
- ☐ Doctorate
- ☐ Other: __________________________

What is your religion?

- ☐ Christian
- ☐ Muslim
- ☐ Jewish
- ☐ Hindu
- ☐ Buddhist
- ☐ Non-religious
- ☐ Rather not say
- ☐ Other: _______________

Ethnicity refers to your family's cultural heritage such as Caucasian, South Asian, South East Asian, East Asian, African, Caribbean, Latino, Middle Eastern, Mixed (please specify), Other, etc. Since people can have more than one race and/or ethnicity, list all that apply.

What is your ethnicity? ___________________________

In what country were you born? ___________________________

In what country do you currently live? ___________________________

How long have you lived in _______? ___________________________

Please read each of the following statements carefully. After you have read all the statements below, place a check by the statements that best depict your exposure to persons with a severe mental illness.

- ☐ I have watched a movie or television show in which a character depicted a person with mental illness.
- ☐ My job involves providing services/treatment for persons with a severe mental illness.
- ☐ I have observed, in passing, a person I believe may have had a severe mental illness.
- ☐ I have observed persons with a severe mental illness on a frequent basis.
- ☐ I have a severe mental illness.
- ☐ I have worked with a person who had a severe mental illness at my place of employment.
- ☐ I have never observed a person that I was aware had a severe mental illness.
- ☐ My job includes providing services to persons with a severe mental illness.
- ☐ A friend of the family has a severe mental illness.
- ☐ I have a relative who has a severe mental illness.
- ☐ I have watched a documentary on the television about severe mental illness.
- ☐ I live with a person who has a severe mental illness.

Next you will see a paragraph describing a person. Please read it carefully and answer the respective questions.

During the last 2 months, one of your friends has changed. Contrary to previous times, he is feeling downcast and sad without any specific reason. He looks concerned and worried. He hardly ever talks and, if he does, he speaks in a low voice about worries concerning the future. Your friend feels useless and a failure. Attempts to cheer him up are not successful. He has lost all his interests. He complains about waking up repeatedly in the middle of the night and about being unable to fall asleep afterwards. In the morning, he feels weary and without energy. He reports to be hardly able to concentrate on his work. Unlike before, every task takes him a long time to do.

What do you think is going on with your friend?

____

To what extent do you think that the following could explain the person's behaviour?
Your friend has …

	Completely explains the behaviour		Not sure		Do not explains the behaviour
problems with their family	☐	☐	☐	☐	☐
brain damage	☐	☐	☐	☐	☐
hormonal imbalance	☐	☐	☐	☐	☐
problems at work	☐	☐	☐	☐	☐
loss of a loved one	☐	☐	☐	☐	☐
experienced a traumatic event	☐	☐	☐	☐	☐
taken drugs	☐	☐	☐	☐	☐

To what extent do you think it would be helpful or harmful for your friend to …?

	Very helpful			Neither Helpful nor harmful			Very harmf ul
See a psychologist	☐	☐	☐	☐	☐	☐	☐
See a GP / doctor	☐	☐	☐	☐	☐	☐	☐
See a psychiatrist	☐	☐	☐	☐	☐	☐	☐
Talk to their children	☐	☐	☐	☐	☐	☐	☐
See a spiritual leader (e.g. priest, imam)	☐	☐	☐	☐	☐	☐	☐
Get some fresh air	☐	☐	☐	☐	☐	☐	☐
Take some vitamins	☐	☐	☐	☐	☐	☐	☐
Talk to their spouse	☐	☐	☐	☐	☐	☐	☐
Go for counseling and/or therapy	☐	☐	☐	☐	☐	☐	☐
Go on a holiday	☐	☐	☐	☐	☐	☐	☐
Talk to a teacher / professor / lecturer	☐	☐	☐	☐	☐	☐	☐
Go to a psychiatric clinic	☐	☐	☐	☐	☐	☐	☐
Talk to friends	☐	☐	☐	☐	☐	☐	☐
Talk to a colleague	☐	☐	☐	☐	☐	☐	☐
Read about mental illness (in a book or on the internet)	☐	☐	☐	☐	☐	☐	☐
Talk to their parents	☐	☐	☐	☐	☐	☐	☐
Take medication (e.g. antidepressants / antipsychotics)	☐	☐	☐	☐	☐	☐	☐
Call a telephone helpline	☐	☐	☐	☐	☐	☐	☐

During the last six months, one of your friends has changed. He withdraws from his co-workers and friends more and more. He keeps out of everybody's way. Contrary to his former habits, he does not take care of his appearance any longer and seems to neglect himself increasingly. He seems to be anxious and agitated. He reports to be convinced that people are able to read other people's thoughts, and that they are also able to influence these thoughts; but he would not yet know who is controlling his thoughts. He even hears these people talking to him and giving him orders. Sometimes, they speak to one another and mock him. In his apartment, the situation is particularly bad. There he feels threatened and terribly scared. He has not been at home for a week and hid in a hotel, which he has not dared to leave.

What do you think is going on with your friend?

____

To what extent do you think that the following could explain the person's behaviour?
Your friend has …

	Completely explains the behaviour		Not sure		Do not explains the behaviour
problems with their family	☐	☐	☐	☐	☐
brain damage	☐	☐	☐	☐	☐
hormonal imbalance	☐	☐	☐	☐	☐
problems at work	☐	☐	☐	☐	☐
loss of a loved one	☐	☐	☐	☐	☐
experienced a traumatic event	☐	☐	☐	☐	☐
taken drugs	☐	☐	☐	☐	☐

To what extent do you think it would be helpful or harmful for your friend to …?

	Very helpful			Neither Helpful nor harmful			Very harmful
See a psychologist	☐	☐	☐	☐	☐	☐	☐
See a GP / doctor	☐	☐	☐	☐	☐	☐	☐
See a psychiatrist	☐	☐	☐	☐	☐	☐	☐
Talk to their children	☐	☐	☐	☐	☐	☐	☐
See a spiritual leader (e.g. priest, imam)	☐	☐	☐	☐	☐	☐	☐
Get some fresh air	☐	☐	☐	☐	☐	☐	☐
Take some vitamins	☐	☐	☐	☐	☐	☐	☐
Talk to their spouse	☐	☐	☐	☐	☐	☐	☐
Go for counseling and/or therapy	☐	☐	☐	☐	☐	☐	☐
Go on a holiday	☐	☐	☐	☐	☐	☐	☐
Talk to a teacher / professor / lecturer	☐	☐	☐	☐	☐	☐	☐
Go to a psychiatric clinic	☐	☐	☐	☐	☐	☐	☐
Talk to friends	☐	☐	☐	☐	☐	☐	☐
Talk to a colleague	☐	☐	☐	☐	☐	☐	☐
Read about mental illness (in a book or on the internet)	☐	☐	☐	☐	☐	☐	☐
Talk to their parents	☐	☐	☐	☐	☐	☐	☐
Take medication (e.g. antidepressants / antipsychotics)	☐	☐	☐	☐	☐	☐	☐
Call a telephone helpline	☐	☐	☐	☐	☐	☐	☐

During the last six months, one of your friends has changed. He presents with extraordinary concern about the safety of his wife and young daughter. He rarely leaves them alone, when away (e.g. at work) he telephones home every hour. He has lost one job because of this. He describes recurrent, unbidden thoughts in which dangerous events befall his family and he is not there to save them. He knows the thoughts are “silly” and they come from his own mind rather than any real danger, but he cannot resist contacting his wife or daughter in some way to be certain they are safe. His wife has arranged to lift the telephone receiver briefly, then hang up, which is usually sufficient to calm his fears for an hour or so. He performs well, and is not particularly perfectionistic, overly conscientious (except with regard to his family’s safety) or rigid.

What do you think is going on with your friend?

____

To what extent do you think that the following could explain the person’s behaviour?
Your friend has …

	Completely explains the behaviour		Not sure		Do not explains the behaviour
problems with their family	☐	☐	☐	☐	☐
brain damage	☐	☐	☐	☐	☐
hormonal imbalance	☐	☐	☐	☐	☐
problems at work	☐	☐	☐	☐	☐
loss of a loved one	☐	☐	☐	☐	☐
experienced a traumatic event	☐	☐	☐	☐	☐
taken drugs	☐	☐	☐	☐	☐

To what extent do you think it would be helpful or harmful for your friend to …?

	Very helpful			Neither Helpful nor harmful			Very harmful
See a psychologist	☐	☐	☐	☐	☐	☐	☐
See a GP / doctor	☐	☐	☐	☐	☐	☐	☐
See a psychiatrist	☐	☐	☐	☐	☐	☐	☐
Talk to their children	☐	☐	☐	☐	☐	☐	☐
See a spiritual leader (e.g. priest, imam)	☐	☐	☐	☐	☐	☐	☐
Get some fresh air	☐	☐	☐	☐	☐	☐	☐
Take some vitamins	☐	☐	☐	☐	☐	☐	☐
Talk to their spouse	☐	☐	☐	☐	☐	☐	☐
Go for counseling and/or therapy	☐	☐	☐	☐	☐	☐	☐
Go on a holiday	☐	☐	☐	☐	☐	☐	☐
Talk to a teacher / professor / lecturer	☐	☐	☐	☐	☐	☐	☐
Go to a psychiatric clinic	☐	☐	☐	☐	☐	☐	☐
Talk to friends	☐	☐	☐	☐	☐	☐	☐
Talk to a colleague	☐	☐	☐	☐	☐	☐	☐
Read about mental illness (in a book or on the internet)	☐	☐	☐	☐	☐	☐	☐
Talk to their parents	☐	☐	☐	☐	☐	☐	☐
Take medication (e.g. antidepressants / antipsychotics)	☐	☐	☐	☐	☐	☐	☐
Call a telephone helpline	☐	☐	☐	☐	☐	☐	☐

What is today’s date? (DD/MM/YY)

Finally, please indicate to what extent you agree or disagree with the following statements.

	Strongly Agree		Neither agree, nor disagree		Strongly disagree
My happiness depends very much on the happiness of those around me.	O	O	O	O	O
I would do what would please my family, even if I detested that activity.	O	O	O	O	O
I usually sacrifice my self-interest for the benefit of my group.	O	O	O	O	O
I enjoy working in situations involving competition with others.	O	O	O	O	O
The well-being of my co-workers is important to me.	O	O	O	O	O
I enjoy being unique and different from others in many ways.	O	O	O	O	O
Children should feel honored if their parents receive a distinguished award.	O	O	O	O	O
I often "do my own thing."	O	O	O	O	O
Competition is the law of nature.	O	O	O	O	O
If a co-worker gets a prize, I would feel proud.	O	O	O	O	O
I am a unique individual.	O	O	O	O	O
I would sacrifice an activity that I enjoy very much if my family did not approve of it.	O	O	O	O	O
Without competition it is not possible to have a good society.	O	O	O	O	O
I feel good when I cooperate with others.	O	O	O	O	O
